# Associations between anxiety and the willingness to be exposed to COVID-19 risk among French young adults during the first pandemic wave

**DOI:** 10.1371/journal.pone.0262368

**Published:** 2022-01-24

**Authors:** Fabrice Etilé, Pierre-Yves Geoffard

**Affiliations:** 1 Paris School of Economics, Paris, France; 2 UMR 1393 Paris-Jourdan Sciences Economiques, Institut national de recherche pour l’agriculture, l’alimentation et l’environnement, Paris, France; 3 UMR Paris-Jourdan Sciences Economiques, Ecole des Hautes Etudes en Sciences Sociales, Paris, France; Bucharest University of Economic Studies, ROMANIA

## Abstract

The COVID-19 outbreak has generated significant uncertainty about the future, especially for young adults. Health and economic threats, as well as more diffuse concerns about the consequences of COVID-19, can trigger feelings of anxiety, leading individuals to adopt uncertainty-reducing behaviours. We tested whether anxiety was associated with an increase in willingness to be exposed to the risk of COVID-19 infection (WiRE) using an online survey administered to 3,110 French individuals aged between 18 and 35 years old during the first pandemic wave and lockdown period (April 2020). Overall, 56.5% of the sample declared a positive WiRE. A one standard deviation increase in psychological state anxiety raised the WiRE by +3.9 pp (95% CI [+1.6, 6.2]). Unemployment was associated with a higher WiRE (+8.2 percentage points (pp); 95% CI [+0.9, 15.4]). One standard deviation increases in perceived hospitalisation risk and in income (+1160€) were associated with a -4.1 pp (95% CI [-6.2, 2.1]) decrease in the WiRE and +2.7 pp increase (95% CI [+1.1, 4.4]), respectively. Overall, our results suggest that both psychological anxiety and the prospect of economic losses can undermine young adults’ adherence to physical distancing recommendations. Public policies targeting young adults must consider both their economic situation and their mental health, and they must use uncertainty-reducing communication strategies.

## Introduction

Even after vaccines become widely available, many countries implement stringent restrictions on individuals’ freedom of movement and socialisation to “flatten” the epidemic waves of the SARS-CoV-2 pandemic. Once a wave subsided, they switch to lighter containment strategies based on physical distancing, population testing, contact tracing, isolation, treatment. While this approach has helped balance social and economic needs within the capacity of the health system [1–4], its effectiveness crucially depends on the willingness of individuals to comply with physical distancing requirements, testing and isolating recommendations, and vaccination when it is available [5–10].

In France, the second, third and fourth waves of COVID-19 infections always started in young adults and spread rapidly to older age groups (see S1 Fig in S1 File). Possible explanations for the reduced prevention efforts among young adults include belief in their low level of health risk, “distancing fatigue,” and the need for social interactions after more than one year of restrictions on social venues. Here, we document an additional explanation: deliberate risk exposure.

Understanding whether and why young adults may be inclined to engage in deliberate risk exposure is important for designing effective public health strategies, especially in the early stages of a pandemic wave, when self- and others-protection efforts are most necessary. During the first lockdown period, some U.S. media reported rumours of “COVID parties,” whereby young individuals would deliberately meet with acquaintances known to be infected and contagious. Even though there is no firm evidence of such practices, these rumours emphasise that intentional self-infection may appear to young healthy adults as a reasonable *individual* strategy of adaptation to their environment.

We hypothesised that feelings of anxiety are one of the factors that can lead young adults to deliberately expose themselves to the risk of contracting COVID-19. We base this hypothesis upon the observation that the COVID-19 outbreak had generated radical uncertainty, triggering feelings of anxiety in many young adults, who faced gloomy economic prospects and physical distancing measures that they may have perceived as a threat to their social bonds and identity [11–13]. Empirical and theoretical works in psychology have largely documented the impact of the subjective experience of uncertainty on the generation of negative emotions, especially anxiety [14]. Heightened anxiety levels pose an adaptative challenge to individuals and thus increase the psychological benefits of uncertainty-reducing actions [15, 16]. Hence, what was possibly at stake, at least among some young adults, was a willingness to eliminate the emotional distress produced by the pandemic as soon as possible. If contracting COVID-19 is perceived as slightly risky in the short term but reduces uncertainty regarding the future, then it may appear preferable to increase one’s risk exposure in order to resolve that uncertainty sooner rather than later [17]. With this theoretical perspective in mind, we propose to examine in this exploratory study whether there is indeed an association between anxiety and exposure to infection risk.

To analyse the associations between anxiety and individual *willingness to risk exposure* (WiRE) to COVID-19, we conducted an online survey in a sample of young French adults aged between 18 and 35 during the first pandemic wave, a period of strict lockdown in the entire country (April 2020). We intended to identify anxiety-related differences in the subjective value of risk exposure after controlling for potential anxiety-related heterogeneity in individual information about the set of possible positive and negative consequences of risk exposure. Therefore, the WiRE question was implemented after having elicited the respondent’s perceptions of COVID-19 health risks, and we deliberately framed our WiRE question to make explicit the possible individual benefits of risk exposure in terms of immunity to COVID-19 and, therefore, reduction in future uncertainty.

We examined whether psychological state anxiety had an independent direct association with WiRE, and whether this association was partly explained by variations in health risk beliefs and economic conditions. Anxiety can have as many sources as there are dimensions of uncertainty, and individuals are likely to differ in their behavioural, cognitive and affective responses to each of these dimensions [18, 19]. Hence, anxiety can vary across individuals according to their economic situation and how they perceive the risk of COVID-19 to be conditional on the information they have. Anxiety can also indirectly affect an individual’s willingness to expose him or herself to risk by shaping his or her risk perceptions and orienting his or her decision-making processes [20, 21]. We therefore introduced income, labour force status and perceived health risks to control for their potential impacts on the association between psychological state anxiety and WiRE.

As a secondary aim of the study, we were also interested in testing whether these control variables were per se significant predictors of WiRE, as they are also potential sources of anxiety. While we expect deliberate risk exposure to decrease with the perceived risk of health dangers, income and labour force status may be ambiguously related to the benefits of risk exposure. The latter depend on the trade-off between the prospect of financial losses in the absence of a return to normal economic life and the expected consequences of infection [22].

In addition to these two categories of anxiety-related motivations—economic incentives and health risk beliefs—compliance with physical distancing norms depends on how much individuals care about the social consequences of their actions [23]. Recent empirical studies reveal that higher levels of social values such as generalised trust, altruism and reciprocity are associated with higher compliance with lockdown measures [24–30]. We specifically focused on generalised trust and reciprocity, as a large body of literature in the social sciences has previously analysed the role of these social values in the emergence of cooperative behaviours at a collective level [31–34], and the social epidemiology literature has also documented their associations with various health outcomes at the individual and community levels [35–37]. We expected that a higher tendency to endorse these social values would be associated with lower WiRE and less sensitivity to anxiety and economic incentives.

While generalised trust and reciprocity refer to the expectation that others will *in general* act in a way that contributes to the common good or will, at least, refrain from harmful actions, individuals may behave differently in personal interactions with particular groups of individuals, depending on whether they feel more or less close to them. We thus also measured psychological closeness with family members, friends, neighbours, and colleagues. In terms of social values, we thus specifically tested whether WiRE was lower in individuals who felt relatively closer to their family than to friends, neighbours, or colleagues and whether this ‘family orientation’ was also a moderating variable in the relationship between anxiety and WiRE.

## Methods

### Setting of the study and statement

We conducted a cross-sectional analysis using data from an online survey that was composed of an experimental investigation on subjective identity, and a general questionnaire including a COVID-19 module. The first author (Fabrice Etilé) had started to design the survey prior to the outbreak as part of a research programme on the relationships between subjective identity and economic preferences in young adults. This research program and the experimental section of the survey are not related to the COVID-19 crisis (see the pre-registration of the experimental hypotheses at https://osf.io/mhty3). After the lockdown and confinement policy measures enacted by the French government on March the 16^th^ of 2020, we decided to add the specific COVID-19 module that was inserted in the questionnaire at a distance from the experimental treatments and measures. Given the time constraints and the crisis context, the hypotheses developed and tested in the present study were not subjected to a specific pre-registration.

The questionnaire was developed using Qualtrics online survey services. The survey respondents were recruited by Qualtrics and paid 4.45 € per completed questionnaire. Qualtrics distributed the survey between April the 10^th^ and April the 20^th^ of 2020. The dataset was made available to us on April the 21^st^ of 2020.

All methods were performed in accordance with relevant French and European guidelines and regulations. The whole survey was approved by the Paris School of Economics IRB (Certificate 2020–009). Although the second author (Pierre-Yves Geoffard) is a member of the Paris School of Economics IRB, he did not participate in reviewing the proposal. The IRB waived the need for informed consent as (i) respondents’ anonymity and RGPD compliance were guaranteed by Qualtrics; (ii) the experimental section of the survey used innocuous priming (iii) respondents were free to opt out of the survey at any moment.

More details on the overall survey structure and content are presented in (S1 File) document Appendix A in S1 File. The data and codes used for producing the results are available at https://osf.io/gesyt/.

### Target population and sample selection

The target sample was specified to include only individuals aged between 18 and 35. The sampling procedure was designed to have equal quotas of women and men and of individuals aged from 18 to 25 (inclusive) and 26 or over. The total analytical sample included 3,100 participants, consisting of 1,570 women and 1,530 men. Details about sample selection can be found in Appendix B in S1 File.

### Target outcome

Our outcome of interest was the self-reported willingness to risk exposure (WiRE) to coronavirus infection. A screening question asked respondents to report whether they thought they had already been infected, with three possible answers: ‘No,’ ‘Yes,’ ‘Maybe, I am not sure.’ Those who answered negatively or were not certain were then asked to report on a 0 to 10 Likert scale their willingness to expose themselves to the risk of infection, where 0 was labelled ‘I would not take any risk’ and 10 as ‘I will take as much risk as I can’. The question was preceded by a short introductory text, based on the state of public knowledge about the disease at the time of the survey [38], emphasising the likely benefit immunity if one recovers from the infection. This was followed by a text warning the respondent that current scientific knowledge indicates that being infected does not guarantee long-term immunity and that the protection of oneself and others requires that contamination risk is minimised by following the government’s recommendations and adopting social distancing measures.

### Anxiety

Psychological anxiety was measured by using the Spielberger state anxiety scale in a 6-item short-form version [39]. Item responses were summed to produce a score from 0 to 18. We did not find clinical cut-offs that would have been validated for the categorisation of young adults facing a pandemic situation as mild, moderately, or severely anxious. We therefore standardised the anxiety score using gender-specific means and standard deviations, as previous studies had shown the impact of gender-specific norms in health-reporting behaviours in France [40].

### Key control variables

Health risk beliefs were measured by asking about respondents’ subjective expectations about the population-level risks and their own risk of COVID-related hospitalisation. We included the measure of population-level risks, and the difference in perceived risks for the population at large and for oneself. This difference may reflect a potential optimism or pessimism bias about the health dangers of contracting COVID-19 [41, 42]. We also included a dummy variable–“COVID negative: unsure”–indicating that the respondent thought that he or she may have already been infected.

Respondents had to report their monthly household income. We adjusted for the number of household units of consumption to produce an equivalised household income variable. We included dummy variables indicating whether the respondent was still working and, if so, whether he or she had arranged to work from home or not. We also collected information on the respondents’ employment status before the crisis and constructed dummy variables indicating whether, before the lockdown, the respondent had a permanent job, had a temporary job, was a student, was self-employed, was unemployed, or was out of the labour force.

Generalised trust was measured through a single question that was drawn from the European Social Survey [43], and had previously been used in studies on health and trust [35]. Respondents had to rate on a 0 to 10 Likert scale whether “most people can be trusted, or one needs to be very careful when dealing with people”. Reciprocity was measured by presenting respondents with a choice scenario in which they had to choose from among a group of gifts of different value what they would give to a stranger who had previously helped them [44].

Psychological closeness with others was measured by using inclusion of other in the self (IOS) scales where individuals report on a 1 to 7 Likert scale how close they feel to another given individual or group of individuals [45]. Respondents had to provide ratings for seven categories of individuals: their partner, their parents, their family, their long-term friends, their work or university friends, people and friends from their neighbourhood, and people they socialise with through organizations. We used the seven ratings to construct two average scores, the first for the respondent’s partner, parents and other family members and the second for the respondent’s other relationships. We hypothesised that risk-taking behaviour would be affected differently if the individual is more family oriented than if he or she is more community (friends, neighbours, colleagues) oriented. Therefore, we defined the *relative* closeness of individuals with their relatives to their closeness with their other acquaintances by dividing the former score by the latter. This ratio measures the relative ‘family orientation’ of individuals. We also computed the average of all ratings to control for inter-individual variability in response styles (see Appendix A.8 in S1 File). The trust, reciprocity and family-orientation variables were all standardised (z-score) by their gender-specific means and standard deviations.

### Statistical analysis

For our main analysis, we fitted ordered logit models (using Stata 16.1) with WiRE as the dependent variable. Our baseline specification included age as a continuous variable and dummy variables for gender, education (in three groups: did not complete high school, completed high school, higher education), partnership status, parental status, income, and labour force status. We adjusted for the confounding effect of risk and time preferences as risk tolerance and impatience have been found to negatively affect prevention efforts [41, 46]. We also controlled for heterogeneity in lockdown conditions: we introduced one dummy variable that indicated whether the respondent was locked down and temporarily not working, and a dummy variable that indicated whether he or she was in lockdown with someone close. These two variables were selected after a specification search that revealed no effect of other characteristics of lockdown conditions (see B.2 in S1 File).

The data were collected in the context of a larger survey that contained experimental priming manipulations on time and identity. Although these manipulations were unrelated to the COVID-19 (see A.1 in S1 File), we controlled for potential survey-specific effects via a set of dummy variables identifying the various experimental groups, with deviation contrast coding to ease the interpretation of the effects on the other dummy variables. These fixed effects were never significant in our regressions. We also included dummies for each quintile of the total survey duration as a means of improving the precision of the estimates and controlling for heterogeneity in response quality.

Starting from a baseline model that included the variables for incentives and the control variables, we successively added health risk beliefs and anxiety. We then tested whether social values and family orientation modulate the effects of incentives and anxiety by introducing appropriate interaction terms.

As the distribution of answers displayed a large grouping on 0, we decided to focus our presentation of the results on the intention to take on at least some risk of exposure, i.e., reporting a positive WiRE. In Section C in S1 File, we report additional results for the probabilities of reporting a WiRE greater than each value of the scale (1, 2,..up to 9). We used the post-estimation Stata command—margins—to report the marginal effect of variables on the probability that an individual’s WiRE would be greater than 0. The results should be interpreted in terms of the change in percentage points for marginal changes of the covariates.

## Results

### Descriptive statistics

The first column of Table 1 displays the average sociodemographic characteristics of the survey respondents. The sample had an almost equal proportion of women to men and individuals aged 25 or under to individuals over 25, as determined by the sampling design. Nearly 95% of the respondents were born in France, 53.7% had attended higher education, and 50.0% of them were living with a partner. While 20.7% had at least one child, only 2.6% were single parents. The average monthly equivalised income was 1,660€, while the median income was lower (1,400€). The income distribution was right-skewed (the skewness equalled 1.66). While more than 25% of respondents had a monthly income lower than 1,000€, another quarter had a monthly income higher than 2000€. About 30% of respondents were still students, 42.7% had a permanent job, 12% had a temporary job, 5.3% were self-employed and 5.6% were unemployed. We discuss the representativeness of this sample later in the article.

**Table 1:** Sample characteristics of survey respondents.

	Full sample	COVID negative		
		*All*	*Women*	*Men*
Number of observations	3,100	2,880 (92.9%)	1,471	1,409
*Socio-demographic characteristics (%)*				
Woman	50.6	51.1	100.0	0.0
18–25 years old	50.0	50.3	55.6	44.7
26–35 years old	50.0	49.7	44.4	55.3
Born in France	95.2	95.3	96.5	94.1
*Schooling (%)*				
Less than Baccalaureate	15.2	15.1	10.7	19.7
Baccalaureate	31.1	31.5	31.1	31.9
Higher education	53.7	53.4	58.2	48.4
*Household structure (%)*				
Has a partner	50.0	49.4	48.5	50.4
Has a child	20.7	20.6	20.3	20.9
Couple with a child	18.0	17.9	16.8	19.1
Single parent family	2.6	2.6	3.5	1.8
Couple without children	32.0	31.5	31.7	31.3
Single or separated	47.3	47.9	48.0	47.8
*Income per UC* (€/month)				
Mean	1,660	1,653	1,554	1,756
Median	1,400	1,400	1,333	1528
Q25 (25^th^ percentile)	933	933	800	1000
Q75 (75^th^ percentile)	3,333	3,333	1,945	2,333
Standard deviation	1,165	1,160	1,093	1,218
*Labour force status (%)*				
Student	30.3	30.6	33.5	27.5
Permanent job	42.7	42.1	37.7	46.6
Temporary job	12.0	12.2	13.9	10.5
Self-employed	5.3	5.3	4.4	6.2
Unemployed	5.6	5.8	6.5	5.0
Out of LF/Other	4.0	4.1	4.1	4.0
*Self-reported COVID status (%)*				
COVID negative: sure	66.3	71.4	70.3	72.5
COVID negative: unsure	26.6	28.6	29.7	27.5
COVID positive	7.1	-	-	-
*Social values and family orientation*				
Trust (0–10 score)	4.18	4.18	4.02	4.34
Reciprocity (Gift in €)	19.19	19.15	18.66	19.67
Family orientation	1.45	1.45	1.46	1.45

Except for income, trust, reciprocity and family orientation, the numbers are sample percentages. Family orientation is calculated by dividing closeness with relatives by closeness with non-relatives.

Nearly 7% of the respondents (280) reported that they believed they had already been infected by COVID-19. Of these ‘COVID-positive’ respondents, only 7.5% provided this answer because they had been tested, 30.5% reported this because a doctor told them they had had it, and 62% based their answer only on symptoms they had had. Of the 2,880 ‘COVID-negative’ respondents, 71.4% were sure that they had not caught it, and the remaining 28.6% were unsure. The second column of Table 1 provides the descriptive statistics for the subsample of ‘COVID-negative’ individuals. Their characteristics are almost identical to those of the full sample. Columns 3 and 4 show the descriptive statistics by gender for the ‘COVID-negative’ respondents. Women were, on average, younger and more educated than men. Women were also more likely to be students than workers, and women in the labour force were more likely to have a temporary job or be unemployed than men in the labour force. This may explain why their equivalised income was lower, with a median of 1,333€ compared to 1,528€ for men. The samples did not significantly differ in social values or family orientation.

**Fig 1:**
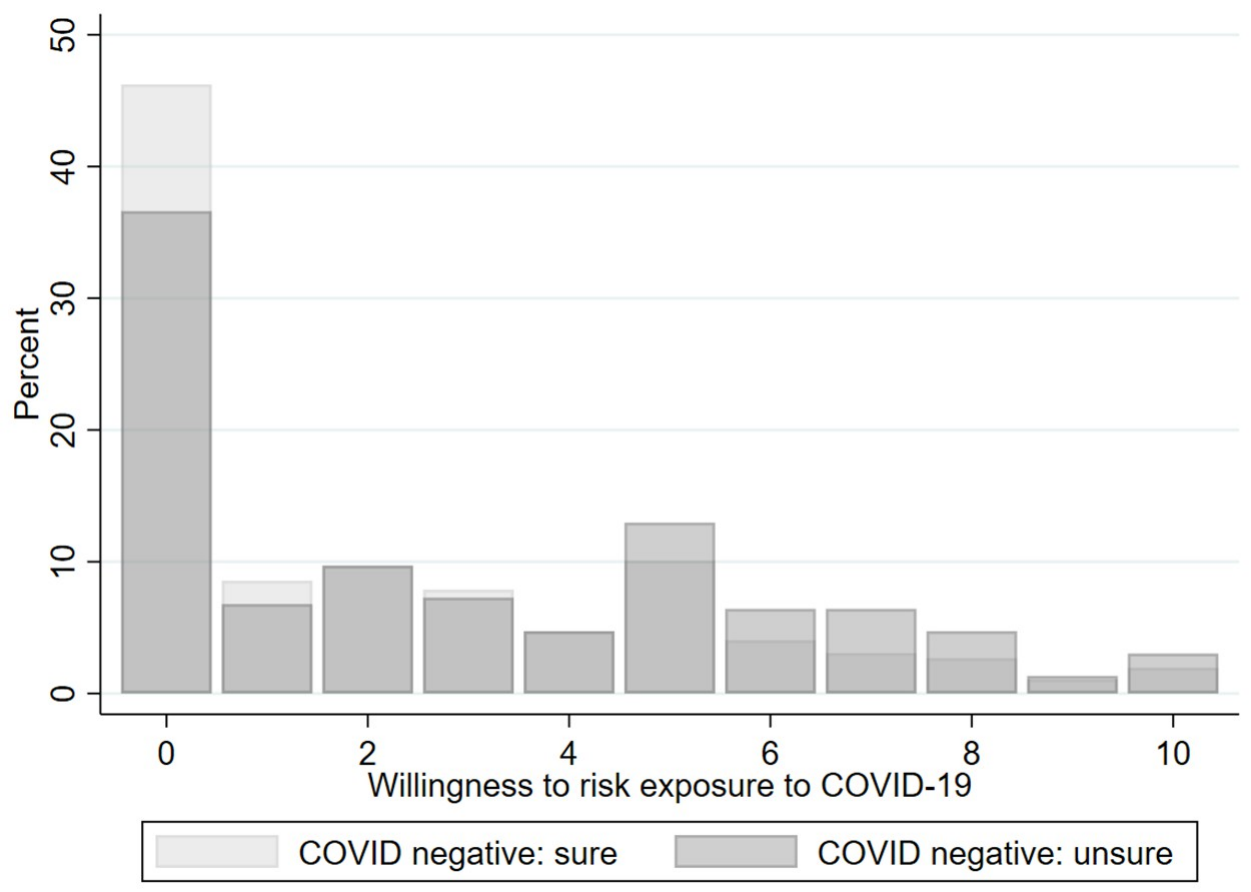
Distribution of willingness to risk exposure by COVID-negative status.

Fig 1 is a histogram of the distribution of the willingness to risk exposure (WiRE), which was self-reported on a 0 (*no risk at all*) to 10 (*maximum risk*) Likert scale. This question was asked only to COVID-negative respondents, and the figure plots distinct histograms for confidently negative vs. less confidently negative respondents. A majority of respondents (56.5% on average) were willing to take at least some risk, with a higher percentage (63.4%) reported by those who were unsure about their negative COVID-19 status.

**Fig 2:**
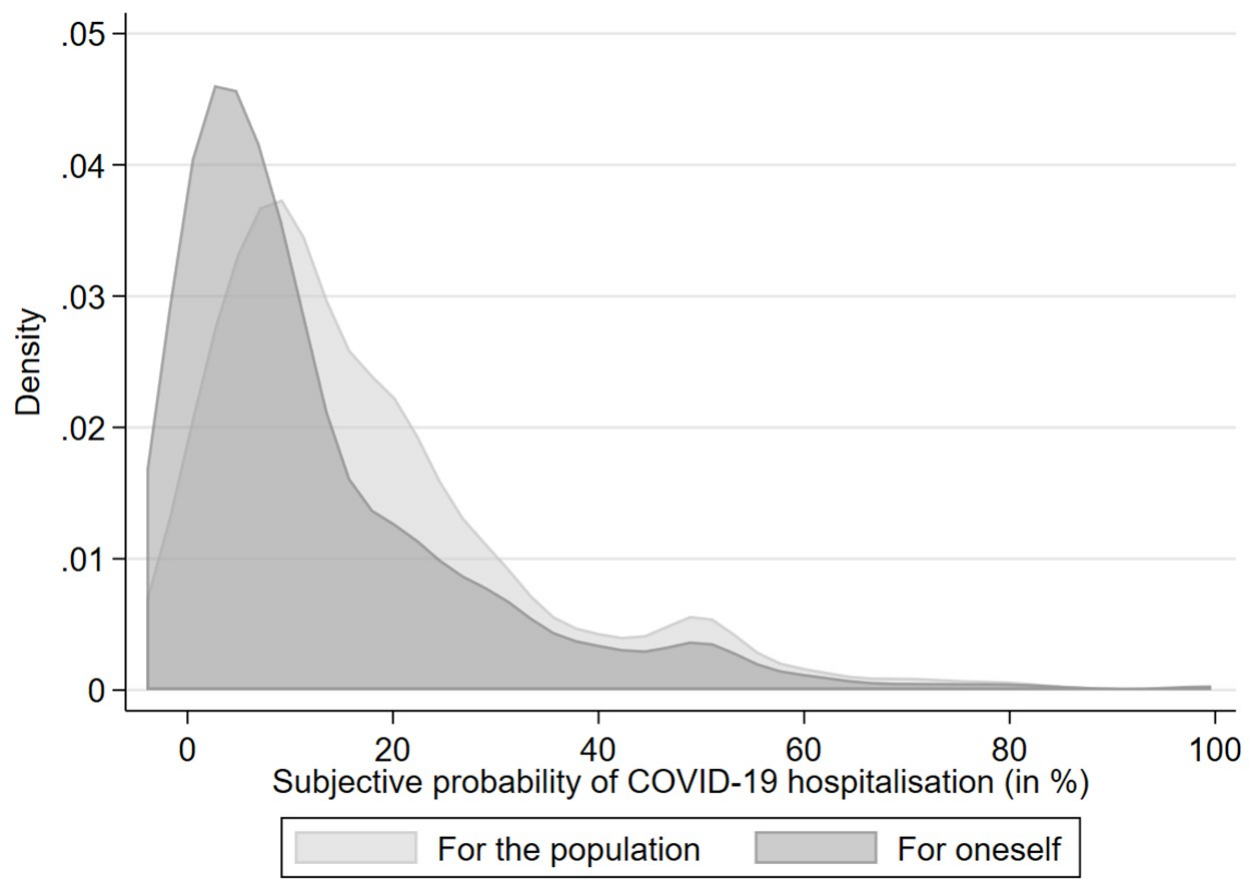
Beliefs about COVID-19.

Fig 2 shows the distributions of subjective beliefs regarding COVID hospitalisation risk for the overall population and for the individuals themselves. More than half of the respondents (55.6%) were more optimistic for themselves than for the population, while less than a quarter (22.9%) were more pessimistic. The average subjective probability of hospitalisation was 17.8% for the population and 12.9% for the individuals themselves. Beyond these differences, the probabilities were significantly correlated, with a Pearson correlation of 0.51.

**Fig 3:**
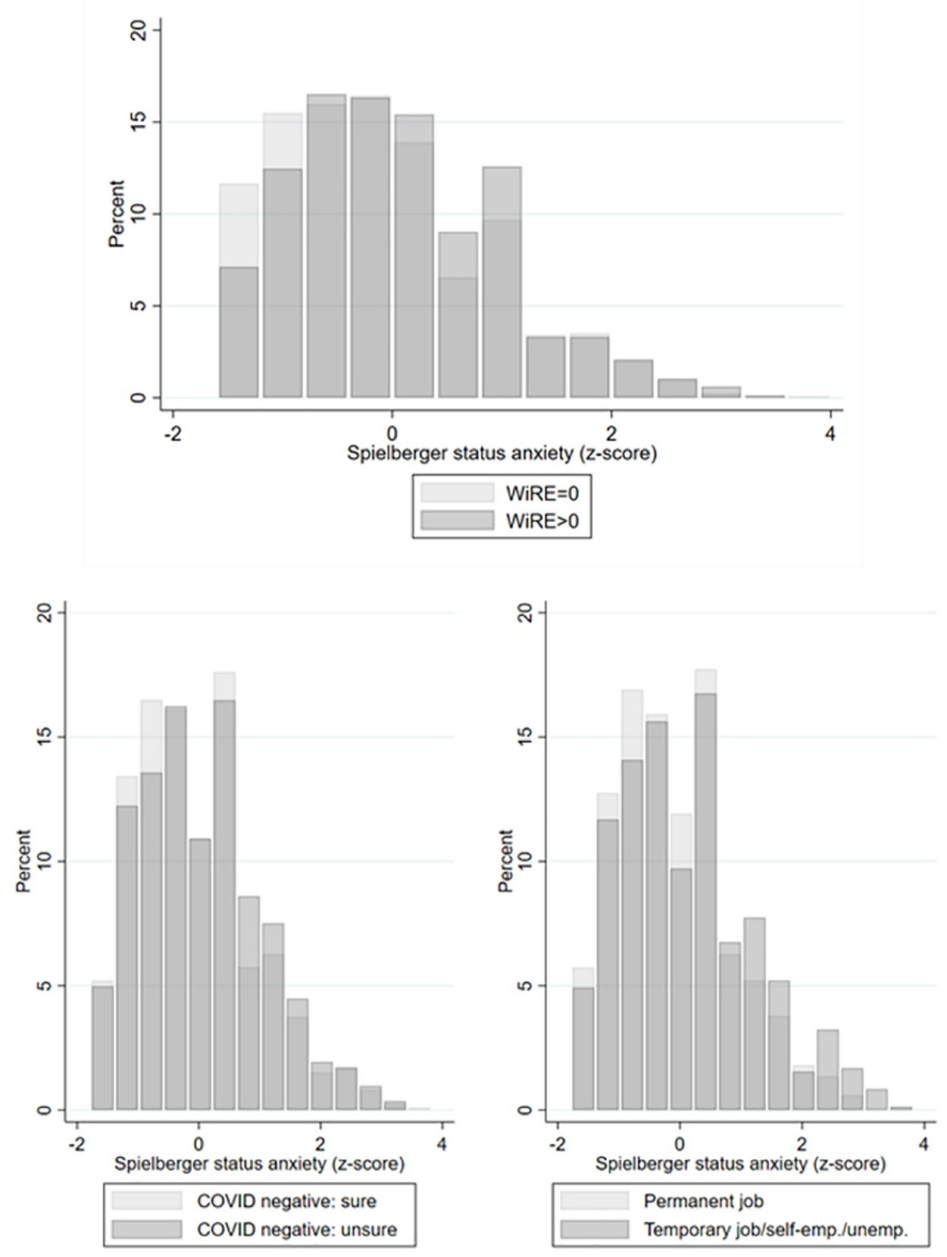
Distribution of status anxiety scores.

Fig 3 displays the distributions of scores on the Spielberger status anxiety scale. The scores were standardised using gender-specific means and standard errors. The distributions were slightly right skewed. We plotted the distributions for respondents with positive vs. null values of WiRE (upper panel), for respondents who were sure vs. unsure about being COVID negative (lower left panel), and for those who had a permanent job vs. had a less secure employment status (lower right). Fig 3 shows that higher anxiety levels are found in individuals with a positive WiRE, in those who thought they might have already been infected, and in those with job insecurity. Taken together, these three plots suggest that economic conditions and health beliefs may partially mediate the association of psychological anxiety with willingness to risk exposure.

### Associations with the WiRE

**Fig 4:**
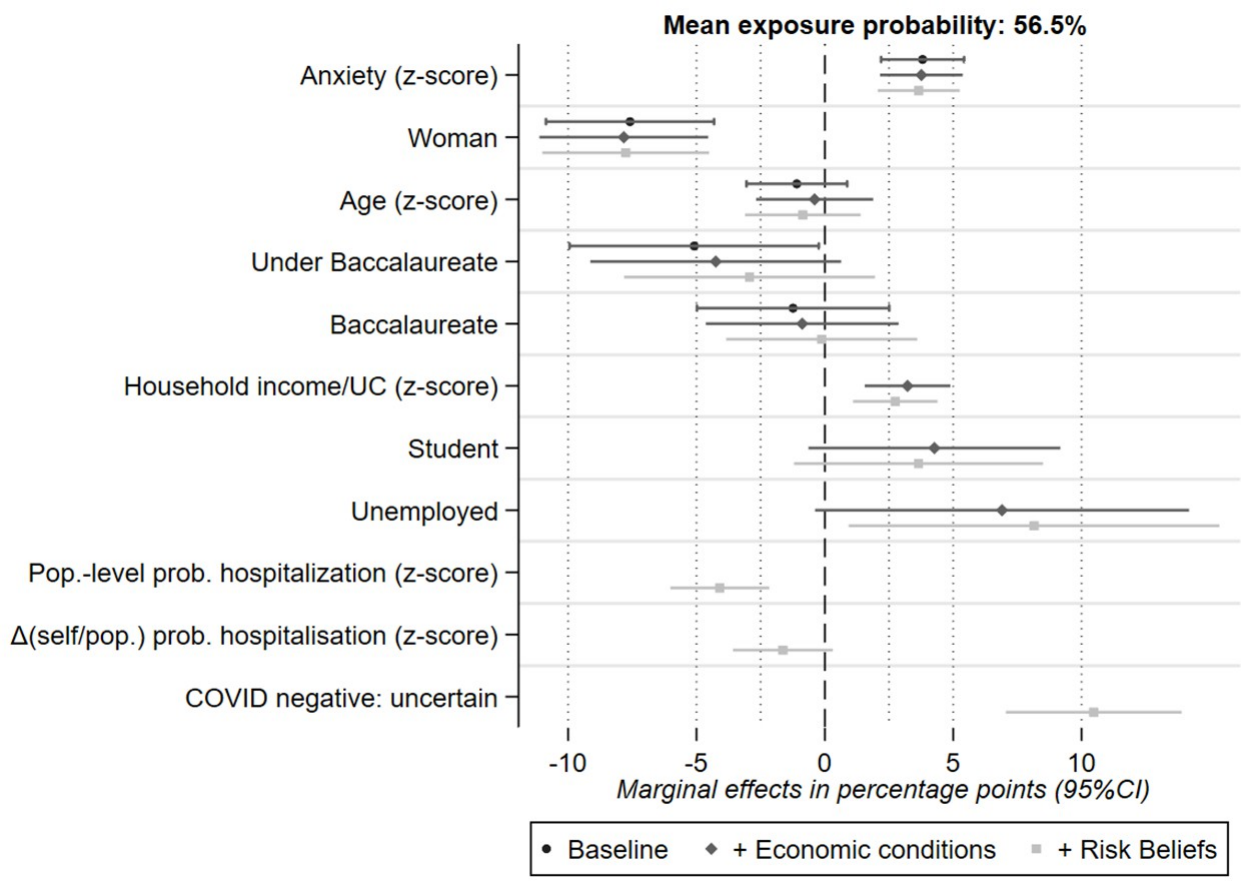
Impact of economic conditions, COVID-19 risk beliefs and anxiety. Full results presented in Table C1 in Appendix C of S1 File. Baseline results in black correspond to Table C1 in S1 File, Specification 2. The results with the addition of health risk beliefs (in dark grey) correspond to Table C1 in S1 File, Specification 3. The results with the addition of health risk beliefs and anxiety (in light grey) correspond to Table C1 in S1 File, Specification 4.

Fig 4 shows the marginal effects of selected covariates on the WiRE. The target outcome is a binary variable for being willing to take at least some risk vs. no risk at all (WiRE>0 *vs*. WiRE = 0). The average unconditional probability of being willing to risk exposure was 56.5%. For the interpretation of the marginal effects of discrete covariates, the reference respondent is a man with a permanent job and a higher education degree who was still working during lockdown. Fig 4 is based on the estimation results reported in Table C1 in S1 File, specification 4.

A one standard deviation increase in anxiety score was associated with a +3.7 pp higher probability of being willing to take risk (95% CI [2.1,5.3]). Adding economic conditions and health risk beliefs as a covariate did not change the estimated effects for psychological anxiety. Hence, psychological anxiety had a large, direct and independent association with WiRE. This result was also left unchanged when we replace the standardised score on the Spielberger state anxiety scale by dummy indicators for being in the second tertile or in the third tertile of the gender-specific distributions of anxiety scores. Being in the second (resp. third) tertile is associated with a +4.6 pp (resp. +8.8 pp) higher probability of being willing to take risk. In a sensitivity analysis, we tested whether this result also holds along the entire distribution of the WiRE, i.e for the probability of reporting a WiRE higher than 2, 3 up to 9. Table C2 in S1 File shows that the association becomes weaker as WiRE increases. A one standard deviation increase in anxiety is associated with a +2.2 pp higher probability of reporting a value higher than 5 (significant at the 5% level), dropping to +1.0 pp for values higher than 7 (significant at the 10% level), and +0.4pp (not significant) thereafter. Hence, the association between anxiety and the WiRE is not driven by large values of WiRE, but rather by positive albeit small to moderate values.

Regarding associations with the control variables, we found that the probability of reporting a positive WiRE was 7.8 percentage points (pp) lower for women than for men after adjusting for a linear age trend, schooling, economic conditions, lockdown status, risk and time preferences, and survey-specific effects. Neither age nor schooling had significant effects, although not having a Baccalaureate’s degree (having a UK A Level or less) was marginally significant at the 10% level in the baseline specification.

Income had a significantly positive effect (p<0.01). Translated into changes in probability, a one standard deviation (1,160€) increases in equivalised income implied that the probability of being willing to take some risk was raised by 2.7 pp (95% CI [1.1,4.4]). Unemployed respondents were also more willing to risk exposure, with a probability 8.2 percentage points higher than that of someone with a permanent job position (95% CI [0.9,15.4]). Associations with other labour force positions (had a temporary job, were self-employed, were not in the labour force) were close to 0 and never significant. Students were also more willing to take risks, with a 4.2 pp higher probability in the baseline specification, which was significant at the 10% level only.

The perception of health risks was negatively associated with the WiRE. A one standard deviation (16.4 pp) increase in the subjective probability that people with COVID-19 are hospitalised decreased the probability of being willing to take risks by -4.1 pp (95% CI [-6.2,-2.1]). We did not detect a specific effect of the difference between beliefs regarding oneself and beliefs regarding the general population. Respondents who were unsure about their COVID-19 status had an 8.5 pp higher probability of being willing to take risks than those who were sure they were COVID-negative (95% CI [4.6,12.5]).

We estimated the full specification on subsamples stratified by gender. The results revealed no significant differences between men and women (see Table C1 in S1 File).

### Social values and family orientation: Direct effects and effect heterogeneity

Do social values and family orientation affect the WiRE and the relationship between the WiRE and anxiety? We started by estimating the direct impact of generalised trust and reciprocity. A one standard deviation increase in the score of generalised trust was associated with a +4.0 pp increase in the probability of being willing to risk exposure (+4.8 for men)–see Fig C2 in S1 File. While high-trust respondents were more willing to risk exposure, we found that reciprocity was negatively and significantly associated with risk exposure for men (-3.7 pp). This association was close to zero and not significant for women. A one standard deviation increase in family orientation had a strong and significant negative impact of -2.2 pp on the probability of risk exposure. The effect was significant and larger for women (-2.9 pp) than for men (-1.7 pp, not significant). The effect of the average psychological closeness rating was negative and not significant.

We finally assessed whether the effect of anxiety, but also economic conditions and health risk beliefs varied across different levels of social values and family orientation with relatives. To this aim, we estimated the full specification with interaction terms between the variables that were significant at the 5% threshold in the main estimates reported in Fig 4 and trust, reciprocity, and family orientation. We then calculated the marginal effects of income, unemployment, health risk beliefs and anxiety at the first and third quartiles of trust, reciprocity and family orientation. The results are displayed in Table 2. Reciprocity had no moderating effects and we do not report the results here. Anxiety displayed a larger association with WiRE for high-trust respondents and those who are less family-oriented. The income effect was slightly larger for low-trust respondents and those who were relatively less family oriented. However, these differences are not statistically significant. Unemployment had a large positive effect for low-trust respondents, with an increase in the WiRE of +12.4 pp. This effect was two and half times lower for high-trust respondents. In contrast, the positive effect of unemployment was higher for more family-oriented respondents (+9.0 pp for highly family-oriented respondents vs. +6.2 pp for low family-oriented respondents). Finally, the effects of health risk beliefs did not vary with trust or family orientation.

**Table 2:** Effect heterogeneity by trust and family orientation.

	Trust		Family orientation	
	Low	High	Low	High
Anxiety	3.423***	4.244***	4.640***	3.473***
(z-score)	(1.049)	(1.030)	(0.938)	(0.860)
Household income/UC	3.226***	2.294**	3.097***	2.334**
(z-score)	(1.131)	(0.933)	(0.957)	(0.910)
Unemployed	12.357***	4.936	6.155	8.977**
	(4.595)	(4.299)	(4.032)	(3.531)
Pop.-level prob. hospitalization	-4.606***	-3.604***	-4.028***	-4.002***
(z-score)	(1.242)	(1.114)	(1.043)	(1.023)
COVID negative: unsure	11.743***	10.470***	8.635***	12.102***
	(2.356)	(2.049)	(2.002)	(1.803)
N observations	2,859	2,859	2,859	2,859

Outcome = probability of taking some level of risk (Likert score on willingness to risk exposure>0). Marginal effects in percentage points estimated from an ordered logit model with willingness to risk exposure as the dependent variable and interaction terms between trust, reciprocity, relative family orientation and the variables in the first column. Low/high trust and (relative) family orientation refer to the 25^th^ and 75^th^ percentile values of these variables.

* *p*<0.1;

** *p*<0.05;

*** *p*<0.01.

## Discussion

As COVID-19 became a global pandemic, most national public health authorities have recommended that individuals engage in various behaviours to protect themselves and others. A large body of research has documented the associations between a number of psychological and socio-economic factors and individual compliance with these recommendations. We here complemented this research by analysing the associations between these factors and individual willingness to deliberately expose oneself to the risk of infection. Deliberate risk exposure may appear to young adults as a reasonable *individual* adaption strategy in face of the economic and existential threats posed by the pandemic. We thus framed our questionnaire in a way that let them to consider both the possible costs and benefits of infection. We found that, in April 2020, a large proportion of young adults were somewhat willing to deliberately expose themselves to the risk of infection. Economic conditions, COVID-19 risks perceptions and psychological anxiety were significantly associated with this attitude.

Psychological (state) anxiety was positively associated with the WiRE. Additional regressions showed that psychological anxiety was higher for individuals with insecure employment and for those who thought they might have already been infected (Table C4 in S1 File). However, including this variable in the model did not change the estimated effects for economic conditions or health risk beliefs. The positive association between anxiety and the WiRE may appear at odds with studies showing that more anxious individuals report a higher compliance with public health recommendations [47, 48]. These studies confirm the general prediction that uncertainty-fuelled anxiety predicts risk avoidance when the choice situation is framed so as to make salient the health risk. However, the research in psychology and neurosciences has found that anxiety can be sometimes associated to increased risk-taking [49–51]. One mechanism is that anxiety increases sensitivity to reward by affecting the neural valuations of choice options [49, 52]. Indeed, our WiRE measurement procedure was intentionally framed in a way that made explicit the possible benefits of infection in terms of immunity for oneself and, therefore, anxiety reduction.

The results also show a negative, albeit small, effect of risk beliefs on the WiRE. A majority of respondents were more optimistic for themselves than for the population. This could be interpreted as an ‘optimism bias’ in risk perceptions [53], but one should also remember that the risks for young adults was already known to be lower than for the general population. Although this bias was associated with a higher WiRE, the association was not significant, while the association between the perceived risks for the population and the WiRE was significantly negative (see the full results, Table C1 in S1 File). These results are in line with analyses of associations between risk perceptions and COVID-19 preventative behaviour in other countries [41, 47, 54]. One policy issue, then, is whether communicating about health risks may effectively alter risk perceptions [55]. We analysed the impact of the daily death toll on respondents’ risk perceptions since we knew when they responded to the survey (the day and hour) and the daily death toll was announced each day at approximately 7:00 pm by the government. In April 2020, over the survey period, the average death toll stood approximately at 800. We found that a reduction in daily deaths by 100 was associated with a decrease in the perceived hospitalisation risk of -0.2, -0.3 pp (Table C4 in S1 File). Being more family-oriented than, for instance, friends-oriented was also negatively related to the WiRE. Caring for one’s relatives is thus an important motivation for avoiding risk exposure. Taken together, these results suggest that more effectively informing young adults about the actual health risks (not just mortality but also morbidity) for themselves and for others, especially older family members, may help reduce their intentional risk exposure.

Unemployment and income were both positively associated with the WiRE. Existing studies have found mixed results about the association between socio-economic status and compliance with public health recommendations regarding COVID-19. For instance, while neighbourhood income was positively correlated with physical distancing in the US, higher SES adults complied less in Switzerland [56, 57]. We interpret our own results as evidence that economic anxiety—the fear of economic losses [58]–partly relates to the opportunity costs of self-protection efforts on the WiRE. High-income respondents may anticipate high income losses if self-protection efforts somehow decrease their productivity. Importantly, the positive income effect cannot be explained by a lower risk aversion in high-income individuals or by a lower discount rate, as we controlled for monetary risk aversion and time preference. To specifically test for the moderating role of economic expectations, we estimated a model where income was interacted with a dummy variable taking a value of 1 if the respondent anticipated a decrease in income at a one-year time horizon and 0 otherwise (see A.5 in S1 File for the definition of the variable). We found a significant positive effect of income for pessimistic respondents only (see Fig C3 in S1 File). Our results also indicated that the effect of unemployment was markedly higher in more family-oriented individuals. This suggests that the same ‘social value’ (caring for one’s relatives) can have very heterogeneous effects, depending on the individual’s specific situation. For family-oriented, unemployed individuals, a pressing need for resources to support one’s family may actually increase risk-taking behaviours. In line with other studies, these results imply that economic compensations may be an effective means of increasing compliance with behavioural measures such as social distancing or quarantine [59, 60].

To the best of our knowledge, this is the first study that examines the determinants of deliberate risk exposure to COVID-19. We think that is not a minor and anecdotal research question, as the COVID-19 pandemic is marked by a flourishing of popular theories, misleading narratives and false beliefs that effectively impact adherence to public health recommendations [61, 62] and to vaccination [63]. Yet, we acknowledge several limitations. First, although we cared for the age-sex representativity of our data, this study lacks socioeconomic representativity. Comparisons with statistics from the French National Statistics Office (INSEE) show that low-income populations are overrepresented, with a poverty rate of approximately 34% for individuals 18–29 years old compared to the national rate of 19.7% in the INSEE data [64, chap. 5.5]. This reflects the lower opportunity cost of time of subjects recruited by online survey companies. This bias in recruitment may be an advantage, as low-income populations are bearing disproportionate health and economic burdens from the epidemic, and they are also more resistant to vaccination [65]. It would nonetheless be informative to test whether our conclusions hold true in a more representative sample.

Second, this study only uses a self-reported and hypothetical measure of willingness to take the risk of becoming infected with Sars-Cov2 in order to be protected from the disease for a period of time. Whether their actual behaviour is based upon such risk attitudes deserves further investigation. In addition, self-reported measures of psychological constructs such as trait anxiety or psychological closeness with others, and self-reported measures of economic characteristics (unemployment, income) may be affected by reporting biases (e.g., social norms). It would thus be worth testing the robustness of our findings with data including more objective measures. For instance, one may examine the spatial and time correlations between the dynamics of some economic measures of economic uncertainty or some clinical indicators of population-level anxiety, and the dynamics of the pandemic.

Third, the study design did not allow us to test directly whether making explicit the idea that contracting COVID-19 could grant immunity to the virus has a causal impact on the WiRE. We have only examined the associations between anxiety and the WiRE *conditional* on this framing. A future replication of this study in the similar context of a pandemic may thus include an experimental random treatment, whereby subjects could be assigned to either a framing or a no-framing condition.

Fourth, the survey was distributed at the very beginning of the pandemic, when vaccines were not available. It would be interesting to reconduct the same survey both in rich countries, where vaccines are available, but a prevention fatigue has emerged (as shown by the street protests against restrictions), and in countries where vaccines are not yet widely available.

Overall, this study examined 18 to 35 years old living in France during the COVID-19 pandemic in April of 2020. We draw from our findings, three important implications for public health communication strategies. First, the motives for self-protection or self-exposure are very heterogeneous, and different categories of young adults are likely to be sensitive to different messages. Second, public communication should avoid increasing perceived uncertainty about the health effects of the virus, or about the social and economic consequences of the outbreak, as this may foster anxiety and therefore increase risk exposure, especially when vaccines are not yet available. The governments’ economic and social measures related to COVID should be complemented by specific actions related to mental health. This would also help increase compliance with public health recommendations. Finally, an emerging concern is that public finances have been severely impacted by the pandemic. The coming pressure for reducing public debts and deficits could have damaging consequences for public health budgets, especially in the mental health sector, that should not be underestimated.

## Supporting information

S1 File (DOCX)
