# Supplementary material for: Associations between anxiety and the willingness to be exposed to COVID-19 risk among French young adults during the first pandemic wave
Source: PLoS One. 2022 Jan 24;17(1):e0262368. doi: 10.1371/journal.pone.0262368 (PMC8786188; doi:10.1371/journal.pone.0262368)
Supplement: S1 File — (DOCX) [file pone.0262368.s001.docx]

**SUPPLEMENTARY INFORMATION FOR:**

**Anxiety increases the willingness to be exposed to COVID-19 risk among young adults in France**

Fabrice Etilé^1,2^*, Pierre-Yves Geoffard^1,3^

^1^ Paris School of Economics, Paris, France.

^2^ UMR 1393 Paris-Jourdan Sciences Economiques, Institut national de recherche pour l’agriculture, l’alimentation et l’environnement, Paris, France

^3^ UMR Paris-Jourdan Sciences Economiques, Ecole des Hautes Etudes en Sciences Sociales, Paris, France

**Correspondence should be addressed to**

*Fabrice Etilé, email: [fabrice.etile@psemail.eu](mailto:fabrice.etile@psemail.eu)

**Fig S1. Weekly trends in the % of positive tests by age cohort (right vertical axis) 01/06-01/11**


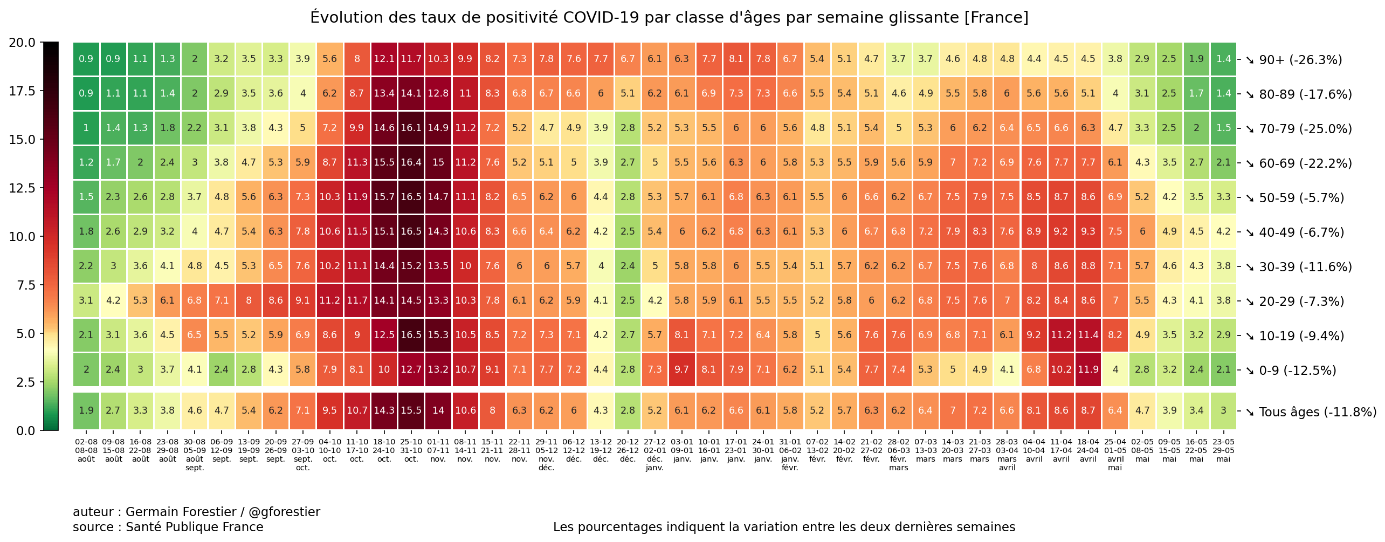


Source: Open data from htttp:/data.gouv.fr; treatment Dr Germain Forestier

https://germain-forestier.info/covid/pos/France-heatmap-pos-semaine.png [retrieved on June 2, 2021]

**A – Development and structure of the survey**

The survey was initially developed by FE as an online experiment aiming at testing various hypotheses about the relationship between subjective identity, and social and time preferences (OSF registration: [osf.io/mhty3](https://osf.io/mhty3)). The development started in January 2020.

Upon the implementation of lockdown measures by the French government (14/03/2020), we designed and added a specific module in the post-experience questionnaire, with the ambition of collecting information on participants’ attitudes and beliefs related to the health and economic crisis.

A.1. Survey flow

1. Questions about gender, age (screening) and place of birth.
2. Experience:
   1. Administration of the French version of the Aspects-of-Identity-Questionnaire-IV (Cheek, Smith et al. 2002, Cheek, Cheek et al. 2018, Yin and Étilé 2019); two Instruction Manipulation Checks (attention checks) are inserted randomly among the items, resulting in screening out upon failure.
   2. Measurement of subjective perceptions of durations.
   3. Experimental treatments (priming manipulations with between-subjects randomization across ten treatment groups).
   4. Measurement of future self-continuity (Ersner-Hershfield, Garton et al. 2009, Hershfield 2011) and psychological closeness with others (Aron, Aron et al. 1992).
   5. Re-exposition to the treatments.
   6. At random, one of social preference monetary tasks OR monetary time discounting tasks (Falk, Becker et al. 2018).
   7. Re-exposition to the treatments.
   8. The other of social preference monetary tasks OR monetary time discounting tasks.
3. Post-experience questionnaire
   1. Monetary measure of willingness-to-take risks (Falk, Becker et al. 2018).
   2. **CoVid-19 Module**
   3. Socio-demographic module: household structure, usual place of residence and housing conditions, labour force status, social class, income, income change expectations.
   4. Additional psychometric module, including inter alia: subjective life expectancy (Dormont, Samson et al. 2018); Likert scales for social preferences (European Social Survey items), and for willingness-to-take risks in general and in the health domain (as in the German Socio-Economic Panel).
   5. Experiment 1: Narrative and associative links ratings
   6. Experiment 2: Various questions related to various issues, including the expectations regarding the political and social situations, and environmental beliefs and attitudes.
   7. Experiment 3: Monetary measure of aversion to ambiguity

A.2. Details on the experimental treatments

As participants started with the experimental part of the questionnaire, it is important to know whether this may have influenced their answers to the COVID-19 questions in the post-experience questionnaire.

The experience used three types of priming manipulations, for a total of ten treatment groups. In a first experimental branch, a specific dimension of identity was made salient by asking respondents to generate freely eight identity aspects that relate to this dimension. Participants had to describe either who they are (Control), or who they are as distinct persons, or who they are in their relationships with close others. In a second experimental branch, a specific dimension of identity was made salient by asking participants to recall childhood memories that seem important to them for understanding who they are as distinct persons, or who they are in their relationships with close others. Subjects in a control group had to remember places they recently visited. In a third experimental branch, we manipulated subjects’ perceptions of the time stability of personal or relational identity in a 2 x 2 between-subjects design.

While none of these experimental treatments is *directly and explicitly* related to the COVID crisis, we are aware that they may have altered respondents’ answers in the post-experience questionnaires. In addition, our empirical analyses use as covariates some of the target outcome variables of the experiments (Monetary discounting and connectedness with others). We therefore included treatment-group fixed-effects in all regressions.

A.3. Details of the COVID-19 module

1. Our COVID-19 module started with two psychometric measure of state anxiety. Importantly, they were administered before we made any mention, explicit or implicit, of the ongoing COVID-10 crisis and the lockdown:
   1. French adaptation of the Spielberger state anxiety scale, 6-items short-form version (Marteau and Bekker 1992, Bruchon-Schweitzer and Paulhan 1993, Gauthier and Bouchard 1993): Je me sens calme – Je suis tendu – Je me sens bouleversé – je suis détendu – Je suis content – Je suis inquiet (Pas du tout/Un peu/Modérément/Beaucoup) *[I feel calm – I am tense – I feel upset – I am relaxed – I feel content – I am worried (Not at all/Somewhat/Moderately/Very much)]*. In the analysis sample (N=3,110), the Cronbach alpha is 0.74. A factor analysis of the polychoric matric of correlations shows a two factor structure after varimax rotation, with the first factor accounting for 71.3% of the variance of the data, while the second factor has an eigenvalue of 0.69, well under the rule-of-thumb threshold of 1. We thus uses the summation of items (1, 4 , 5 reverse-coded) as a scale measuring state anxiety.
   2. An 11-points Likert anxiety scale asking respondents to “tell what is their level of anxiety, worry of nervousness compared to usual”, where 0 means “I am not more anxious, worried or nervous than usual” and 10 means “I am much more anxious, worried or nervous than usual”. There is a correlation of Rho = 0.56 between this Likert scale and the Spielberger state anxiety scale.
2. A first series of questions intended to measure the respondent’s material condition of living during the lockdown
   1. ‘Lockdown status’: (i) at home not working; (ii) at home, working remotely; (iii) still working away from home.
   2. ‘Lockdown mates’: living with family or friends, alone or other; responding in a calm environment.
   3. Place of residence: (i) big city; (ii) suburbs of a big city; (iii) mid-sized city; (iv) small town or rural area.
   4. Type of housing: (i) 1 bedroom flat; (ii) 2-3 bedrooms flat; (iii) 4-bedrooms flat or more, or a house without a garden; (iv) house with a garden.
3. A second series of questions was related to more specific aspects of participants’ life and feelings under the lockdown – we do not use these variables in the current analysis:
   1. We asked them to rank a number of things to what they miss most to what they miss least: i) do shopping; (ii) physical contact with people; (iii) be free of movements (iv) go in social places (v) go on work or study place (vi) be physically with friends and family. A seventh item was left open to be filled and ranked among others by the subject.
   2. We then asked them to rate on 5-points Likert scale the degree to which they changed their behaviours in various domains: use of social networks, reading books, physical exercise, think about one’s life, worrying, think about social/political/environmental involvements, making plans for one’s professional life or studies, making plans for one’s personal life, making plans regarding one’s friends or one’s family.
4. The last series of questions was related to health beliefs and attitudes. It is detailed in the next subsection.

A.4. Details on the measurement of health beliefs and attitudes

We here present the questions we asked, with the exact wording in French and English reported in Table A.1.

We started by measuring respondents’ subjective probabilities regarding the population-level COVID-related risks, with three risk levels: (i) mild symptoms ; (ii) severe symptoms (fever during more than one week, persistent coughing, tiredness, feeling like a weight on chests...); (iii) Hospitalisation.

We then asked them to self-report their infection status, with three possibilities: No, Maybe, Yes (Covid-19 subjective status question). Those who answered positively (about 7%) had then to tell how they knew they had been infected: (i) because they had COVID symptoms; (ii) because a doctor told them they had it; (iii) because they had been hospitalised or tested.

Those who answered negatively or were not sure were asked to provide their subjective probabilities of suffering from mild symptoms, from severe symptoms or be hospitalized if they were infected. They further had to reveal their intentions regarding whether they would be willing to take the risk of being infected, after a short introductory text emphasising the likely immunity benefits of having recovered from a virus infection. The willingness to deliberately take the risk of being infected was measured on an 11-points Likert scale. The wording of this text was intentionally based on the most common and popular belief regarding the acquisition of an immune defence against a virus.

As this question may arguably contribute to the diffusion of false information, we provided them with the following text (with a warning in red colour) immediately after:

“Be careful, this last question does not mean that being infected with the coronavirus guarantees long-term immunity. Scientists have little knowledge of the virus at present, and the only effective way to protect against it over the very long term is to administer a vaccine, which could be available within nine to eighteen months. In the meantime, for your protection and the protection of others, the risks of contamination should be minimized by following the government's recommendations and adopting social distancing measures.”

**Table A.1**

| **Variable** | **Original wording (French)** | **Translation (English)** |
| --- | --- | --- |
| Subjective expectation regarding the population-level probability of hospitalization | 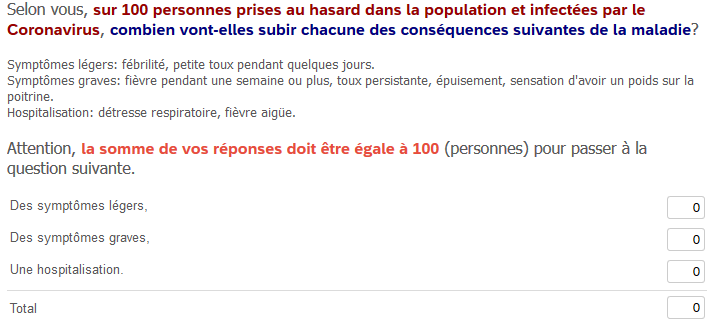 | How many out of 100 randomly selected people in the population infected with Coronavirus would you expect to experience each of the following consequences of the disease?  Mild symptoms: fever, mild cough for a few days.  Severe symptoms: fever for a week or more, persistent cough, exhaustion, feeling of weight on the chest.  Hospitalization: Respiratory distress, acute fever.  Please note that the sum of your answers must equal 100 (people) to move on to the next question. |
| COVID-19 subjective status | 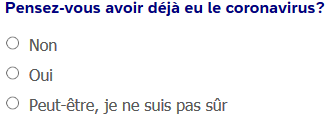 | Do you think you have ever had coronavirus?  No / Yes / Maybe, I am not certain. |
| *For those answering “Yes” or “Maybe, I am not certain” to the previous question:* | | |
| Subjective expectation regarding the probability of hospitalization for oneself | 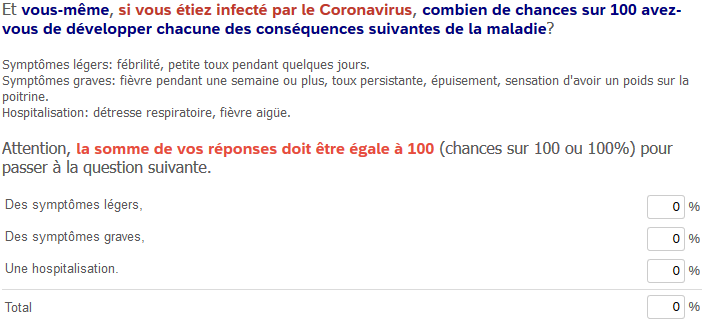 | And yourself, if you were infected with the Coronavirus, what is your 100 per cent chance of developing each of the following consequences of the disease?  Mild symptoms: fever, mild cough for a few days.  Severe symptoms: fever for a week or more, persistent cough, exhaustion, feeling heavy on the chest.  Hospitalization: Respiratory distress, acute fever.  Please note that the sum of your answers must equal 100 (odds of 100 or 100%) to move on to the next question. |
| Deliberate risk exposure | 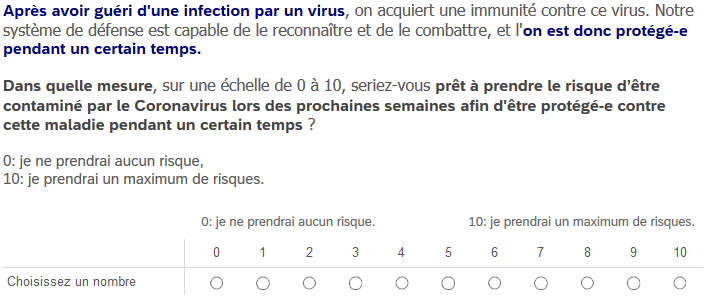 | Once you have recovered from a virus infection, you acquire immunity to that virus. Our defensive system can recognize and fight it, so we are protected for a period of time.  On a scale of 0 to 10, how willing would you be to take the risk of becoming infected with Coronavirus in the next few weeks in order to be protected from this disease for a period of time?  0: I would not take any risk,  10: I will take as big risks as I can. |
| Warning message | 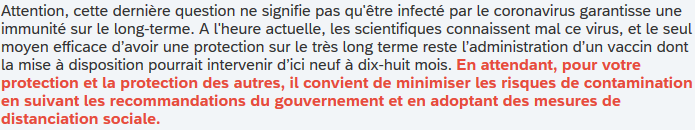 | Be careful, this last question does not mean that being infected with the coronavirus guarantees long-term immunity. Scientists have little knowledge of the virus at present, and the only effective way to protect against it over the very long term is to administer a vaccine, which could be available within nine to eighteen months. In the meantime, for your protection and the protection of others, the risks of contamination should be minimized by following the government's recommendations and adopting social distancing measures. |
| *For those answering “No” to the COVID-19 status question* | | |
| Source of information regarding COVID-19 status | 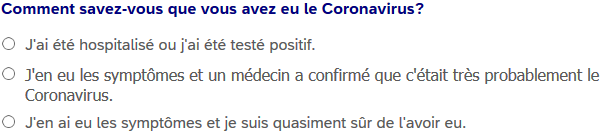 | How do you know you have had Coronavirus?  I have been hospitalized and tested positive  I have had the symptoms and a doctor confirmed it was highly likely that I had the Coronavirus  I have had the symptoms and I am almost sure I caught it. |

A.5. Measuring economic conditions

Respondents were asked to report their monthly household income in € using the following bracket categories: <700; [700,900[; [900;1,100[; [1,100;1,300[; [1,300;1,500[; [1,500;1,800[; 1,800;2,200[; [2,200;2,500[; [2,500;3,000[; [3,000;4,000[; >4,000 €. A continuous income variable was derived by taking the mid-interval values, and 6,000€ for individuals declaring earnings over 6,000€/month.

We also asked them to report their household structure, *assuming that an individual living by his parents is as a single person household*: single or living in partnership; no child, one child, two children or more. We thus computed the household units of consumption (UC), with the convention that one child is associated to 0.3 UC, and the partner counts for 0.5 UC. Following OECD guidelines, this is an approximation as children over 14 years-old should count for 0.5 UC, and households with more than three children are counted as if they had two children. However, given the age range of subjects, we are confident that few of them were in this situation.

A continuous income per unit of consumption variable was then calculated.

Labour force status was measured with 6 categories: self-employed, permanent job, temporary job, unemployed, student, out of the labour force or other.

Expectations regarding income changes were measured by asking respondents to state the probabilities that in one year their income will (one probability per event): (i) have increased a lot (ii) have increased (iii) be about the same (iv) have decreased (v) have decreased a lot. The sum of probabilities had to equal 100 for being granted access to the next question.

Appendix A.6 – Economic preferences

*Risk preference questions*

A sequence of five interdependent questions asked respondents to choose between a sure payment of 300€ and a draw with equal probability to get 0 or some amount *x* that was varied across the sequence to converge to some interval of indifference [x_1_, x_2_] between the sure payment and the lottery. There are 32 such intervals and they are contiguous, which provides a score of risk loving: the lower [x_1_, x_2_] is, the less willing the respondent is to take risks. This score was z-standardized in the analysis.

*Time preference questions*

A sequence of four interdependent questions asked respondents to choose between a payment of 200€ in one year and some amount *x* today. The initial choice was made with *x*=100€ and then *x* was varied across the sequence depending on previous answers, to converge toward an interval of indifference [x_1_, x_2_] between the sooner and the later payment. We calculated the one-year discount factor as 0.5*(x_1_/200+ x_2_/200).

Appendix A.7 – Social values

*Generalised trust* (ESS item)

It is common to measure generalised trust by asking people *“Generally speaking, would you say that most people can be trusted, or that you can’t be too careful in dealing with people?”*.

We used the following French version of this question :


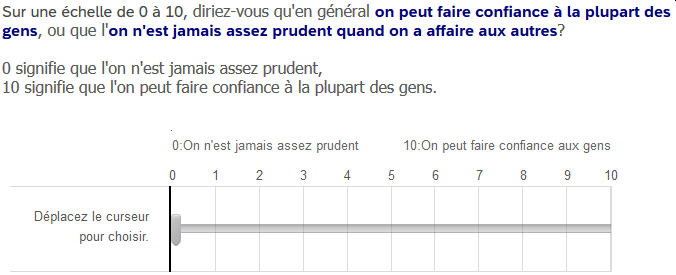


*Reciprocity*

Reciprocity was measured by placing the respondent in a hypothetical situation of social exchange. We used a scenario proposed by Falk, Becker et al. (2018):

*“Please think about what you would do in the following situation. You are in an area you are not familiar with, and you realize you lost your way. You ask a stranger for directions. The stranger offers to take you to your destination. Helping you costs the stranger about 20 Euro in total. However, the stranger says he or she does not want any money from you. You have six presents with you. The cheapest present costs 5 Euro, the most expensive one costs 30 Euro. Do you give one of the presents to the stranger as a “thank-you”-gift? If so, which present do you give to the stranger? No present/ The present worth 5: 10 / 15 / 20 / 25 / 30 €”*

We used the following French version of this question:


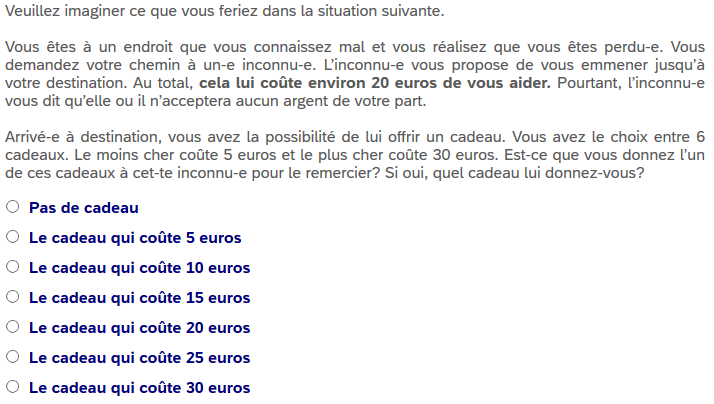


We did not constrain respondents to answer to this question during the “soft-launch” period of the survey. This error produced 24 missing values.

Appendix A.8 – Closeness with others

Closeness with others was measured by asking respondents to rate on a 1-7 scale their degree of psychological connectedness with other categories of individuals. The scores were anchored using circles, following the Inclusion of Other in Self framing proposed by Aron, Aron et al. (1992)


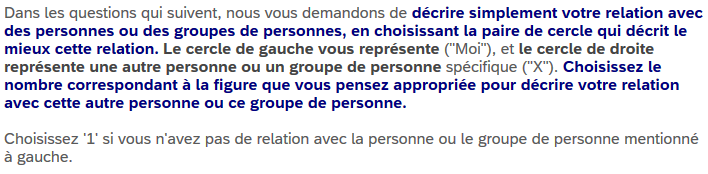


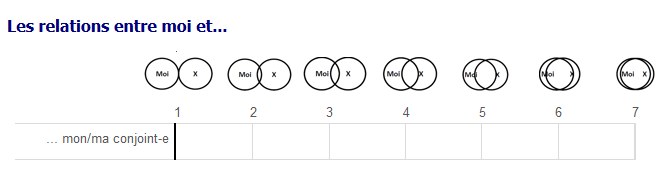


The distribution of answers revealed that subjects feel closer to their family than to their friends, colleagues or other acquaintances:


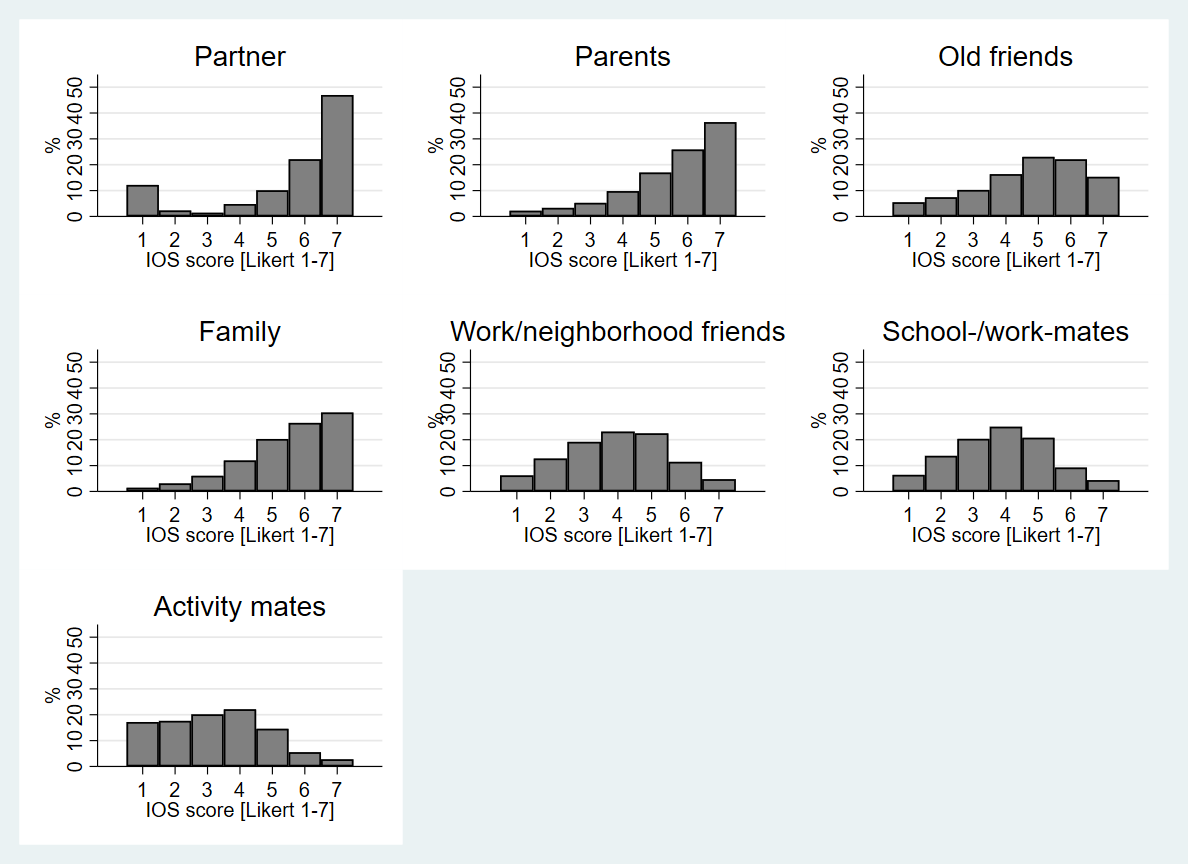


A rating of 1 has to be interpreted cautiously, as it may just mean that the category is not relevant, e.g. the respondent does not have a partner. The average scores measuring closeness with relatives and non-relatives can thus be calculated by excluding or not these ratings. These two options produce measures that are very correlated – see the table below.

| Closeness |  | **Mean** | **Std. dev.** | **Correlations** |
| --- | --- | --- | --- | --- |
| *with relatives* | *excluding the “1”s* | 5.75 | 1.09 | 0.88 |
| *with relatives* | *keeping the “1”s* | 5.53 | 1.24 |  |
| *with non-relatives* | *excluding the “1”s* | 4.32 | 1.13 | 0.93 |
| *with non-relatives* | *keeping the “1”s* | 4.18 | 1.20 |  |

**B – Descriptive Statistics**

The raw data are available in French (original data file) and English (variable labels translated) as Raw_Qualtrics_Data_EN.csv at <https://osf.io/gesyt/>

The replication do files (Stata) are available at <https://osf.io/gesyt/>

B-1 Sample Selection

- Respondents were forced to answer to all questions throughout the survey and knew they were going to be paid only upon full completion of the questionnaire. We therefore did not have to exclude participants with missing values on outcome variables or covariates.
- The COVID questionnaire also included a branching question asking participants whether they were still under lockdown of whether the lockdown measures had been lifted. We further excluded 10 participants who answered the lockdown had been lifted, since this was not the case.
- The distribution of interview duration has the following statistical characteristics:

Mean: 32 min 30 s - Median: 27 min 22 s

5^th^ percentile: 16 min 56 s

10^th^ percentile: 19 min 8 s

20^th^ percentile: 21 min 28 s

40^th^ percentile: 25 min 32 s

60^th^ percentile: 29 min 43 s

80^th^ percentile: 36 min 42 s

90^th^ percentile: 44 min 24 s

95^th^ percentile: 55 min 5 s

Preliminary regressions showed that WiRE decreases with interview duration, even after controlling for time and risk preferences. We therefore decided to control for quintiles of interview duration in all regressions.

B-2 Impact of lockdown conditions – specification search

As we initially thought that lockdown conditions could have a large impact on willingness to risk exposure, we included several questions in the survey to measure whether respondents were still working or working at home, where they were locked down, with whom, in which type of housing, and even whether they were able to view landscapes, stars or large horizons from where they lived (See Appendix A.3). Table B.1 below reports some descriptive statistics.

Table B2 shows how self-reported COVID status is correlated with employment status during locked down. The respondents that were still working outside were less likely to report being surely COVID-negative than the respondents that were remaining inactive at home (58.5% vs. 70.5%, two-sample test of proportions rejects equality at any significance level). They were also significantly more likely to report a COVID-positive status (9.4% vs. 5.7%, p-value<0.01). While we may attribute this to pure beliefs updating – those who kept working outside were objectively more exposed -, Table B.2 also shows that respondents working at home were more likely to think they had been contaminated than those who were not working. Hence, beliefs are in part related to the activity status during the lock down period. One explanatory mechanism for this is that individuals have *motivated* beliefs about their COVID status, i.e. they thought they were already contaminated in order to be less anxious. Such motivated beliefs were formed in the service of higher goals, such as keeping on working. Although we cannot rigorously test this explanation with our data, Table B3 proposes some suggestive evidence that those who kept working were less anxious than those who remained inactive. Tables B2 and B3 eventually illustrate the complexity of relationships between lockdown conditions, health beliefs and anxiety.

Finally, Table B4 presents a set of regressions where the dependent variable is a binary indicator for having a positive willingness to risk exposure. Marginal effects (in percentage points) are reported. The results clearly show that after adjustment for demographic and economic characteristics of respondents, two variables only are significant; being locked down at home *and* inactive, and being locked down with someone close

**Table B1. Lockdown conditions (N=3,100)**

|  | % |
| --- | --- |
| *Lockdown status* |  |
| At home, not working | 48.40 |
| At home, working | 32.81 |
| Work away from home | 18.78 |
|  |  |
| Locked down with someone close | 83.47 |
| Does not live at usual place of residence | 12.08 |
| *Place of residence* |  |
| Large/metropolitan city | 22.19 |
| Suburbs | 13.78 |
| Middle-sized city | 17.67 |
| Small city/rural | 46.35 |
| *Housing conditions* |  |
| Single bedroom flat or other | 6.63 |
| Two- or three-bedrooms flat | 30.31 |
| Four-bedrooms flat or house without garden | 16.70 |
| House with garden | 46.35 |
|  |  |

**Table B2. Self-reported COVID status by lockdown status**

| % | COVID negative: sure | COVID negative: unsure | COVID positive |
| --- | --- | --- | --- |
| *Lockdown status* |  |  |  |
| At home, not working | 70.50 | 23.82 | 5.68 |
| At home, working | 64.88 | 27.32 | 7.80 |
| Work away from home | 58.29 | 32.33 | 9.38 |
| *Average* | *66.29* | *26.61* | *7.10* |

Reading note: 70.5% of respondents at home, inactive reported they were surely COVID negative.

**Table B3. Anxiety by lockdown status**

| % | Anxiety : 1^st^ tercile | 2^nd^ tercile | 3^rd^ tercile |
| --- | --- | --- | --- |
| *Lockdown status* |  |  |  |
| At home, not working | 35.79 | 31.60 | 32.61 |
| At home, working | 41.85 | 30.44 | 27.71 |
| Work away from home | 42.21 | 28.98 | 28.81 |
| *Average* | *39.03* | *30.71* | *30.26* |

Reading note: 35.79% of respondents at home, inactive had a score on the Spielberger status anxiety questionnaire that placed the

**Table B4. Impact of lockdown conditions**

| **Sample** | **COVID negative** | | | **COVID negative** | |
| --- | --- | --- | --- | --- | --- |
|  |  | | | **Females** | **Males** |
| *Lockdown status, Reference: work away from home* | | | | | |
| At home, not working | -6.642*** | -6.825*** | -7.607*** | -8.609** | -6.717** |
|  | (2.233) | (2.246) | (2.351) | (3.347) | (3.290) |
| At home, working | -0.652 | -1.070 | -3.495 | -3.996 | -4.529 |
|  | (2.351) | (2.388) | (2.478) | (3.572) | (3.426) |
|  |  |  |  |  |  |
| Locked down with someone close |  | -3.538 | -5.510** | -2.356 | -6.767** |
|  |  | (2.406) | (2.613) | (3.969) | (3.449) |
| Does not live at usual place of residence |  | 3.067 | 3.475 | 7.014** | -0.379 |
|  |  | (2.521) | (2.563) | (3.407) | (3.991) |
| *Place of residence (Reference: small city or rural area)* | | | | | |
| Large/metropolitan city |  | 2.766 | 2.208 | 2.066 | 1.584 |
|  |  | (2.379) | (2.403) | (3.491) | (3.315) |
| Suburbs |  | 0.730 | -0.285 | -3.586 | 1.474 |
|  |  | (2.628) | (2.641) | (3.763) | (3.683) |
| Middle-sized city |  | 0.966 | 0.764 | 1.929 | -1.708 |
|  |  | (2.426) | (2.433) | (3.493) | (3.388) |
| *Housing conditions, Reference: House with garden* | | | | | |
| Single bedroom flat or other |  | -1.674 | -0.576 | 8.497* | -9.574* |
|  |  | (3.781) | (3.817) | (5.075) | (5.788) |
| Two- or three-bedrooms flat |  | 1.929 | 3.655 | 4.757 | 3.363 |
|  |  | (2.199) | (2.241) | (3.191) | (3.144) |
| Four-bedrooms flat or house without garden |  | 0.187 | 0.618 | 1.980 | -0.748 |
|  |  | (2.499) | (2.501) | (3.755) | (3.308) |
| *Control variables* | | | | | |
| Age, gender, schooling, hhold struct. | No | No | Yes | Yes | Yes |
| Income and labour force status | No | No | Yes | Yes | Yes |
| N observations | 2,880 | 2,880 | 2,880 | 1,471 | 1,409 |

Note: Outcome = probability to take some risk (Likert score>0). * *p*<0.1; ** *p*<0.05; *** *p*<0.01. Reference category: higher educated male with permanent job, working outside or at home during lockdown. Additional control variables in all regressions: quintiles of survey duration, treatment group fixed effects, risk and time preferences.

**Table B5. COVID risk beliefs by self-reported COVID status**

| ***Severity of symptoms*** | **Mild** | **Strong** | **Hospitalisation** |
| --- | --- | --- | --- |
| *Beliefs about population risk (probabilities in %)- N=3,100* | | | |
| COVID negative: sure | 52.84 | 23.98 | 18.52 |
| COVID negative: unsure | 58.89 | 22.41 | 16.80 |
| COVID positive | 60.25 | 21.93 | 14.97 |
| Average | 54.98 | 23.42 | 17.81 |
| *Beliefs about risks for oneself (probabilities in %) – N=2,880* | | | |
| COVID negative: sure | 65.34 | 20.84 | 13.82 |
| COVID negative: unsure | 70.60 | 18.62 | 10.77 |
| Average | 66.85 | 20.20 | 12.95 |
| *Difference between risk for oneself and population risk – N=2,880* | | | |
| COVID negative: sure | 12.49 | -3.14 | -4.70 |
| COVID negative: unsure | 11.71 | -3.78 | -6.03 |
| Average | 12.27 | -3.32 | -5.08 |

# Appendix C – Main and additional results

**Table C1. Main results**

| **Specification** | **1** | **2** | **3** | **4** | **4-Females** | | **4-Males** | |  |  |
| --- | --- | --- | --- | --- | --- | --- | --- | --- | --- | --- |
| **Anxiety** | | | | | | | | |  |  |
| Spielberger status anxiety (z-score) | 3.721*** | 3.809*** | 3.761*** | 3.658*** | 3.429*** | | 3.913*** | |  |  |
|  | (0.825) | (0.826) | (0.823) | (0.815) | (1.147) | | (1.164) | |  |  |
| **Socio-demographic characteristics** | | | | | | | | |  |  |
| Woman | -7.995*** | -7.587*** | -7.831*** | -7.756*** |  |  | | |  |  |
|  | (1.670) | (1.670) | (1.677) | (1.657) |  |  | | |  |  |
| Age (z-score) | -0.665 | -1.091 | -0.397 | -0.857 | -2.178 | 1.044 | | | |  |
|  | (0.971) | (1.000) | (1.163) | (1.149) | (1.748) | (1.551) | | | |  |
| *Schooling. Reference: Higher education.* | | | | | | | | | | |
| Under Baccalaureate | -6.508*** | -5.088** | -4.249* | -2.932 | -3.645 | | -2.614 | |  |  |
|  | (2.447) | (2.477) | (2.493) | (2.494) | (4.182) | | (3.149) | |  |  |
| Baccalaureate | -2.165 | -1.236 | -0.881 | -0.120 | 2.308 | | -2.100 | |  |  |
|  | (1.896) | (1.910) | (1.916) | (1.899) | (2.693) | | (2.702) | |  |  |
| *Household structure* | | | | | | | | | | |
| Has a partner | 0.584 | 0.822 | 2.261 | 1.785 | -0.646 | | 3.924 | |  |  |
|  | (1.788) | (1.875) | (1.910) | (1.886) | (2.617) | | (2.738) | |  |  |
| Has a child | -2.795 | -2.204 | -0.920 | -0.049 | -3.216 | | 2.468 | |  |  |
|  | (2.413) | (2.432) | (2.444) | (2.419) | (3.774) | | (3.230) | |  |  |
| **Equivalised income & Labour force status.** *Reference: permanent job.* | | | | | | | |  |  |  |
| Households income/UC (z-score) |  |  | 3.223*** | 2.745*** | 2.280* | | 3.216*** | |  |  |
|  |  |  | (0.851) | (0.840) | (1.176) | | (1.199) | |  |  |
| Students |  |  | 4.268* | 3.649 | 0.869 | | 6.457* | |  |  |
|  |  |  | (2.504) | (2.476) | (3.551) | | (3.486) | |  |  |
| Temporary Job |  |  | -0.612 | -0.438 | -1.077 | | -1.132 | |  |  |
|  |  |  | (2.709) | (2.672) | (3.717) | | (3.866) | |  |  |
| Self-employed |  |  | 0.623 | 0.069 | 3.818 | | -2.125 | |  |  |
|  |  |  | (3.781) | (3.745) | (5.969) | | (4.764) | |  |  |
| Unemployed |  |  | 6.903* | 8.152** | 7.560 | | 9.491* | |  |  |
|  |  |  | (3.717) | (3.684) | (5.017) | | (5.551) | |  |  |
| Out of LF/Other |  |  | -1.149 | 0.084 | 0.943 | | 1.337 | |  |  |
|  |  |  | (4.505) | (4.446) | (6.448) | | (6.213) | |  |  |
| At home, not working |  | -5.879*** | -5.326*** | -4.720*** | -5.820** | | -3.364 | |  |  |
|  |  | (1.671) | (1.743) | (1.725) | (2.478) | | (2.417) | |  |  |
| Locked down with someone close |  | -2.851 | -4.942** | -4.808** | -3.682 | | -5.107 | |  |  |
|  |  | (2.364) | (2.417) | (2.388) | (3.562) | | (3.220) | |  |  |
| **Health risk beliefs** | | | | | | | |  |  |  |
| Beliefs about population risk of hospitalisation | | | | | | | | | | |
| Pr(hospitalisation) – z-score |  |  |  | -4.093*** | -4.443*** | | -3.686*** | |  |  |
|  |  |  |  | (0.982) | (1.417) | | (1.373) | |  |  |
| *Difference between hospitalisation risk for oneself and for the population* | | | | | | | | | | |
| ΔProbabilities – z-score |  |  |  | -1.632* | -2.296 | | -0.918 | |  |  |
|  |  |  |  | (0.992) | (1.416) | | (1.400) | |  |  |
| COVID negative: unsure *vs.* sure |  |  |  | 10.476*** | 11.894*** | | 9.328*** | |  |  |
|  |  |  |  | (1.746) | (2.471) | | (2.472) | |  |  |
| N observations | 2,880 | 2,880 | 2,880 | 2,880 | 1,471 | | 1,409 | |  |  |

Notes: Outcome = probability that WiRE>0. Marginal effects in percentage points estimated from an ordered logit model, with WiRE measured on a 0-10 Likert scale as dependent variable. * *p*<0.1; ** *p*<0.05; *** *p*<0.01. Reference category: higher educated male with permanent job, working away from home or at home during lockdown. Additional control variables in all regressions: quintiles of survey duration, treatment group fixed effects, risk and time preferences.

Specification 1: estimated on full sample, sociodemographic and economic variables only.

Specification 2: Specification 1 + control for lockdown conditions (only variables that are significant in Table B8 below)

Specification 3: Specification 2 + control for heterogeneity in beliefs regarding the hospitalization risk and one’s own COVID status

Specification 4: Specification 3 + Spielberger status anxiety scale (gender specific z-score).

Specification 5: Specification 4, but marginal effects estimated using a logit model for willingness to risk exposure>0 (all positive scores were grouped).

Specification 4-Females: Specification 4, sample restricted to females.

Specification 4-Males: Specification 4, sample restricted to males.

| **Probability WiRE >** |  | **>1** | **>2** | **>3** | **>4** | **>5** | **>6** | **>7** | **>8** | **>9** |
| --- | --- | --- | --- | --- | --- | --- | --- | --- | --- | --- |
| Spielberger state anxiety, z-score |  | 4.110*** | 3.417*** | 3.893*** | 3.311*** | 2.221*** | 1.587** | 1.044* | 0.552 | 0.414 |
|  |  | (0.925) | (0.930) | (0.902) | (0.852) | (0.813) | (0.654) | (0.566) | (0.462) | (0.331) |
| Household Income/UC (z-score) |  | 1.893* | 2.311** | 2.377** | 1.925** | 2.814*** | 2.499*** | 1.714*** | 1.400*** | 0.768** |
|  |  | (0.978) | (0.971) | (0.928) | (0.862) | (0.801) | (0.614) | (0.529) | (0.428) | (0.319) |
| Student |  | 2.968 | 1.795 | 3.112 | 3.195 | 2.743 | 4.837** | 2.080 | 1.328 | -0.243 |
|  |  | (2.807) | (2.835) | (2.769) | (2.624) | (2.499) | (2.036) | (1.767) | (1.439) | (1.047) |
| Unemployed |  | 9.074** | 7.112* | 3.923 | 4.968 | 2.697 | 5.430* | 4.596* | 3.332* | 2.231* |
|  |  | (4.210) | (4.242) | (4.202) | (3.971) | (3.855) | (3.082) | (2.574) | (2.002) | (1.308) |
| Pr(hospitalisation) – z-score |  | -4.121*** | -3.970*** | -3.128*** | -3.414*** | -3.312*** | -3.483*** | -2.310*** | -1.529** | -0.799* |
|  |  | (1.066) | (1.098) | (1.094) | (1.068) | (1.038) | (0.930) | (0.816) | (0.655) | (0.475) |
| ΔProbabilities – z-score |  | -1.332 | -0.966 | -1.503 | -1.869* | -1.805* | -2.039** | -1.730** | -0.263 | -0.258 |
|  |  | (1.065) | (1.093) | (1.088) | (1.061) | (1.031) | (0.918) | (0.809) | (0.607) | (0.444) |
| COVID negative: unsure *vs.* sure |  | 8.525*** | 10.444*** | 10.276*** | 10.814*** | 10.283*** | 7.290*** | 5.365*** | 2.894*** | 1.161 |
|  |  | (2.014) | (2.000) | (1.921) | (1.787) | (1.684) | (1.358) | (1.176) | (0.969) | (0.716) |
| N observations |  | 2,880 | 2,880 | 2,880 | 2,880 | 2,880 | 2,880 | 2,880 | 2,880 | 2,880 |

**Table C2. Non linearities**

Notes: Outcomes = probability that WiRE>X where X is indicated in the column. Marginal effects in percentage points estimated from a logit model. * *p*<0.1; ** *p*<0.05; *** *p*<0.01. Specification 4 as in Table C.1.

**Fig C1. Impact of economic conditions, health risk beliefs and anxiety by gender**

**
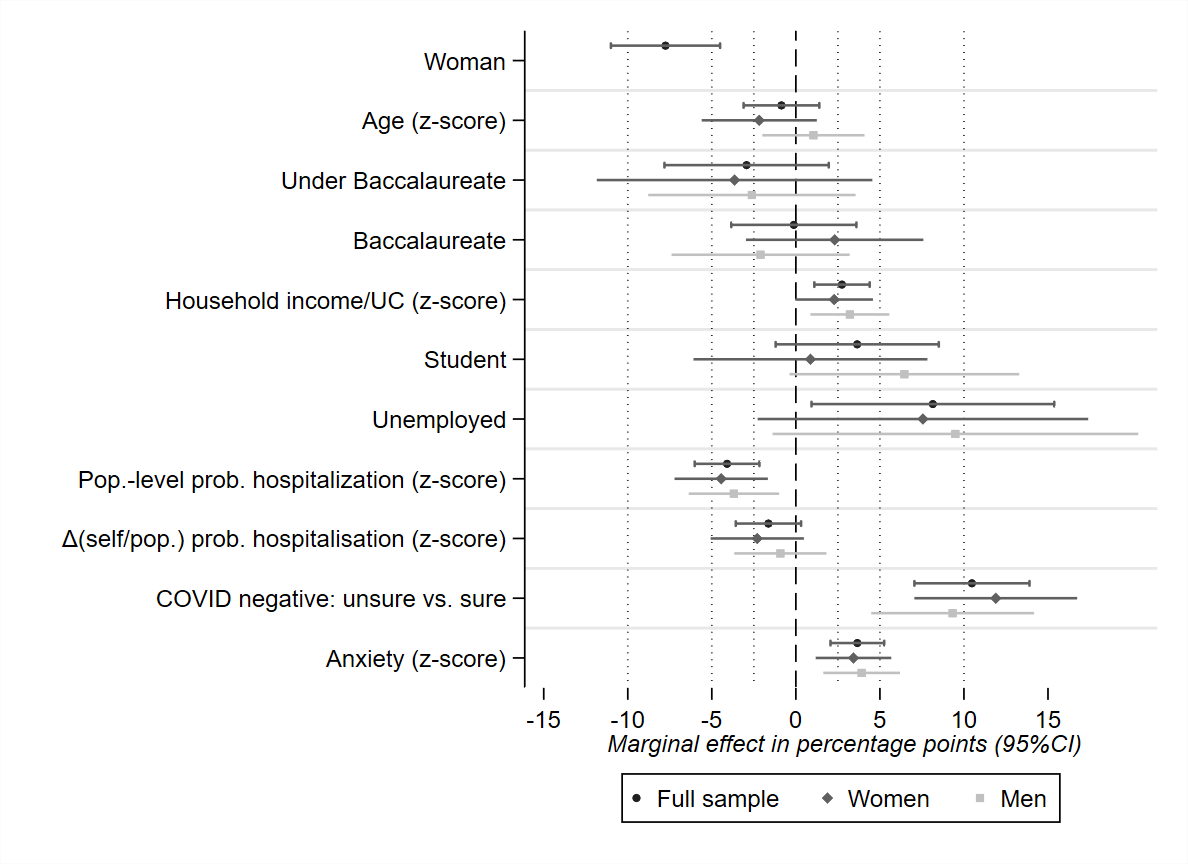
**

**Fig C2. Direct effect of social values and family orientation**

**
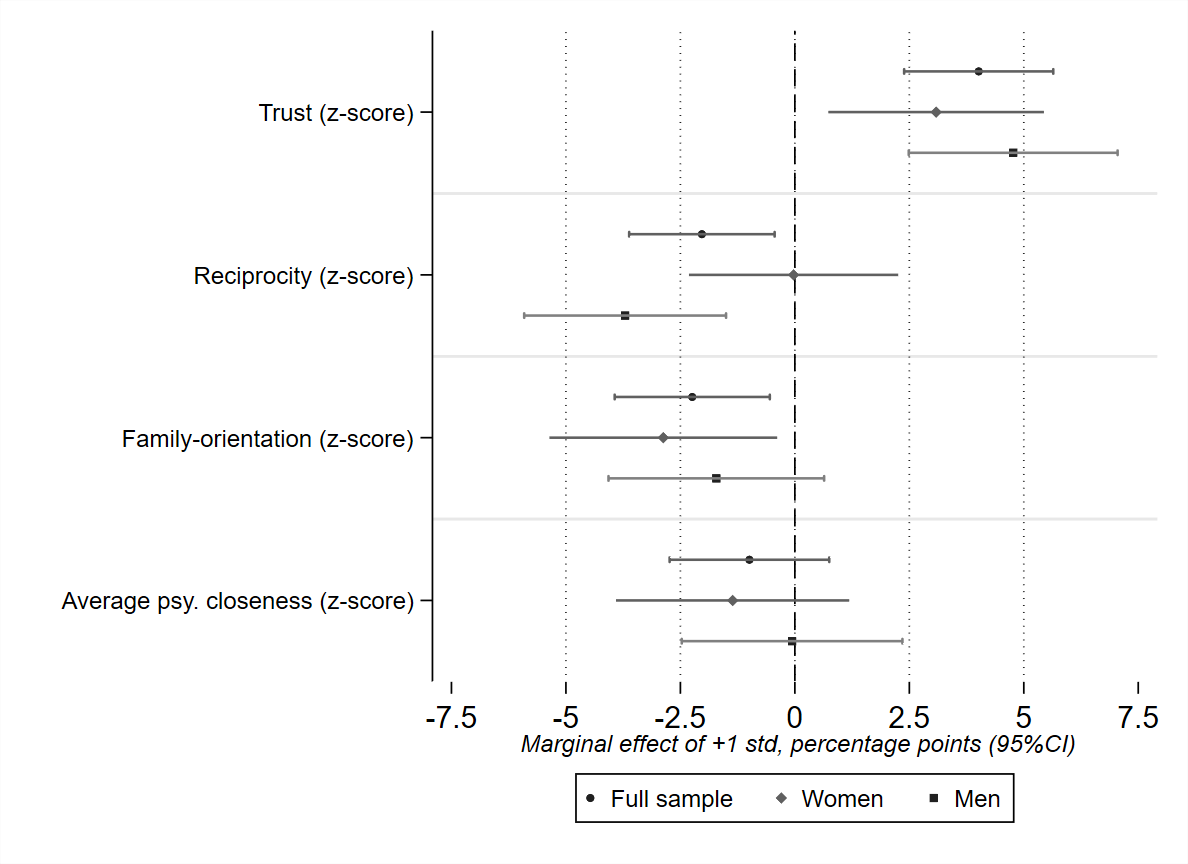
**

**Table C3. Direct effect of social values and family orientation**

| **Specification** | **4** | **4 - Women** | **4 - Men** |
| --- | --- | --- | --- |
| Trust (z-score) | 4.015*** | 3.085** | 4.768*** |
|  | (0.831) | (1.202) | (1.164) |
| Reciprocity (z-score) | -2.030** | -0.028 | -3.707*** |
|  | (0.811) | (1.166) | (1.125) |
| Family orientation (z-score) | -2.241*** | -2.873** | -1.716 |
|  | (0.864) | (1.269) | (1.201) |
| Average psychological closeness (z-score) | -0.992 | -1.358 | -0.060 |
|  | (0.889) | (1.300) | (1.229) |
| N observations | 2,859 | 1,456 | 1,403 |

Notes: Specification 4 as in Table C1. Outcome variable = probability that WiRE>0. Marginal effects in percentage points estimated from an ordered logit model using WiRE as dependent variable. * *p*<0.1; ** *p*<0.05; *** *p*<0.01. Twenty-one observations dropped from the sample due to missing values on the reciprocation measure.

**Figure C3. Heterogeneity in income effects**

**
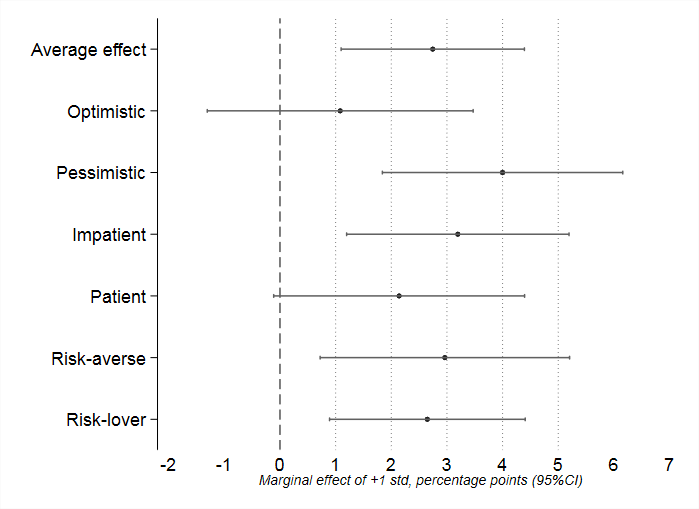
**

**Table C4. Determinants of beliefs about the risk of hospitalisation**

| **Specification** | **1** | **1** | **1** | **2** | **2-F** | **2-M** |
| --- | --- | --- | --- | --- | --- | --- |
| *Beliefs about the risk of COVID hospitalisation for the general population, in percentage points* | | | | | | |
| Death toll (100s) | 0.353*** | 0.350*** | 0.265** | 0.256** | 0.124 | 0.263 |
|  | (0.110) | (0.110) | (0.126) | (0.122) | (0.199) | (0.161) |
| COVID negative: unsure |  | -1.620** | -1.164* | -1.923*** | -1.276 | -2.458*** |
|  |  | (0.670) | (0.668) | (0.639) | (0.867) | (0.948) |
| COVID positive |  | -3.219*** | -2.863** | -2.342** | -2.963* | -1.708 |
|  |  | (1.161) | (1.152) | (1.128) | (1.670) | (1.546) |
| Household income/UC |  |  | -0.638** | -1.050*** | -1.233*** | -0.899** |
| (z-score) |  |  | (0.306) | (0.292) | (0.404) | (0.427) |
| Unemployed |  |  | 0.082 | 0.126 | 1.848 | -2.513 |
|  |  |  | (1.357) | (1.321) | (1.771) | (2.006) |
| At home, not working |  |  | 0.321 | 1.127* | 0.680 | 1.450* |
|  |  |  | (0.624) | (0.601) | (0.834) | (0.879) |
| N observations | 3,100 | 3,100 | 3,100 | 2,912 | 1,480 | 1,432 |
| *Beliefs about one’s own risk of COVID hospitalisation, in percentage points* | | | | | | |
| Death toll (100s) | 0.250** | 0.259** | 0.240* | 0.335** | 0.259 | 0.276 |
|  | (0.111) | (0.111) | (0.127) | (0.133) | (0.213) | (0.178) |
| COVID negative: unsure |  | -2.907*** | -2.395*** | -2.722*** | -1.944** | -3.632*** |
|  |  | (0.657) | (0.651) | (0.682) | (0.917) | (1.019) |
| Household income/UC |  |  | -1.326*** | -1.467*** | -1.576*** | -1.357*** |
| (z-score) |  |  | (0.310) | (0.328) | (0.447) | (0.486) |
| Unemployed |  |  | -0.483 | 0.293 | 0.541 | -0.542 |
|  |  |  | (1.360) | (1.449) | (1.897) | (2.258) |
| At home, not working |  |  | 1.192* | 1.065 | 0.745 | 1.463 |
|  |  |  | (0.630) | (0.660) | (0.910) | (0.966) |
| N observation | 2,880 | 2,880 | 2,880 | 2,398 | 1,221 | 1,177 |
| *Impact on the difference between beliefs about the risk for oneself and the population risk* | | | | | | |
| Death toll (100s) | -0.104 | -0.100 | -0.052 | 0.024 | 0.061 | -0.002 |
|  | (0.112) | (0.112) | (0.130) | (0.125) | (0.214) | (0.157) |
| COVID negative: unsure |  | -1.279* | -1.212* | -0.094 | -0.169 | -0.155 |
|  |  | (0.664) | (0.667) | (0.637) | (0.916) | (0.886) |
| Household income/UC |  |  | -0.690** | -0.588* | -0.383 | -0.730* |
| (z-score) |  |  | (0.318) | (0.306) | (0.445) | (0.424) |
| Unemployed |  |  | -0.212 | 0.202 | -1.417 | 2.453 |
|  |  |  | (1.393) | (1.361) | (1.891) | (2.009) |
| At home, not working |  |  | 0.863 | -0.246 | -0.152 | -0.031 |
|  |  |  | (0.645) | (0.616) | (0.910) | (0.842) |
| N observations | 2,880 | 2,880 | 2,880 | 2,335 | 1,195 | 1,140 |
| *Control variables* |  |  |  |  |  |  |
| Day linear trend | No | No | Yes | Yes | Yes | Yes |
| Additional control | No | No | Yes | Yes | Yes | Yes |

Note: the coefficient must be interpreted in percentage points. * *p*<0.1; ** *p*<0.05; *** *p*<0.01 ; OLS regressions; specification 1: full sample; specification 2 : drops observation with reported probability of hospitalisation risk equals to 0% or 100%; specification 2-F: females; specification 2-M: males. Additional control variables: gender, age, education, has a partner, has a child, other labour force statuses, locked down with some close. The death toll daily counts were divided by 100.

**Table C5. Determinants of anxiety**

| **Sample** | **Full** | **Full** | **Full** | **Females** | **Males** | |
| --- | --- | --- | --- | --- | --- | --- |
| Woman | 0.018 | 0.009 | 0.004 |  |  | |
|  | (0.037) | (0.037) | (0.037) |  |  | |
| Age (z-score) | 0.018 | 0.015 | 0.014 | 0.013 | 0.014 | |
|  | (0.021) | (0.026) | (0.026) | (0.039) | (0.035) | |
| *Schooling (ref ; Higher education)* | | | | | | |
| Under Baccalaureate | 0.136** | 0.094* | 0.082 | 0.041 | 0.135* | |
|  | (0.053) | (0.055) | (0.055) | (0.088) | (0.071) | |
| Baccalaureate | -0.003 | -0.013 | -0.018 | -0.045 | 0.015 | |
|  | (0.042) | (0.042) | (0.042) | (0.060) | (0.061) | |
| *Household structure* | | | | | | |
| Has a partner | -0.108*** | -0.058 | -0.063 | -0.074 | -0.071 | |
|  | (0.039) | (0.042) | (0.042) | (0.058) | (0.061) | |
| Has a child | -0.031 | -0.007 | -0.001 | 0.001 | 0.006 | |
|  | (0.053) | (0.054) | (0.054) | (0.081) | (0.073) | |
|  |  |  |  |  |  | |
| **Income, Labour force status before and during lockdown**  **(***Ref: permanent job*, still working)*.* | | | | | | |
| Household income/UC (z-score) |  | -0.015 | -0.017 | 0.005 | -0.035 | |
|  |  | (0.019) | (0.019) | (0.027) | (0.027) | |
| Student |  | 0.082 | 0.085 | 0.087 | 0.090 | |
|  |  | (0.055) | (0.055) | (0.079) | (0.079) | |
| **Temporary Job** |  | **0.125**** | **0.126**** | **0.001** | **0.271***** | |
|  |  | **(0.060)** | **(0.060)** | **(0.083)** | **(0.088)** | |
| **Self-employed** |  | **0.197**** | **0.203**** | **0.171** | **0.215**** | |
|  |  | **(0.083)** | **(0.082)** | **(0.127)** | **(0.109)** | |
| **Unemployed** |  | **0.277***** | **0.292***** | **0.194*** | **0.395***** | |
|  |  | **(0.084)** | **(0.084)** | **(0.115)** | **(0.124)** | |
| Out of LF/Other |  | 0.020 | 0.014 | -0.007 | -0.000 | |
|  |  | (0.096) | (0.096) | (0.139) | (0.137) | |
| **At home, not working** |  | **0.076**** | **0.082**** | **0.144***** | **0.014** | |
|  |  | **(0.038)** | **(0.038)** | **(0.055)** | **(0.055)** | |
| **Locked down with someone close** |  | **-0.159***** | **-0.160***** | **-0.150*** | **-0.165**** | |
|  |  | **(0.054)** | **(0.054)** | **(0.080)** | **(0.073)** | |
| **Health risk beliefs** | | | | | | |
| *Beliefs about population risk of hospitalisation* | | | | | |  |
| Pr(hospitalisation) |  |  | 0.002** | 0.002 | 0.003* | |
|  |  |  | (0.001) | (0.002) | (0.002) | |
| COVID negative: unsure |  |  | 0.130*** | 0.182*** | 0.074 | |
|  |  |  | (0.041) | (0.058) | (0.059) | |
| COVID positive |  |  | 0.190*** | 0.098 | 0.256*** | |
|  |  |  | (0.071) | (0.106) | (0.096) | |
| N observations | 3,100 | 3,100 | 3,100 | 1,570 | 1,530 | |

Notes: Dependent variable: Score on Spielberger status anxiety scale (z-scored by gender). * *p*<0.1; ** *p*<0.05; *** *p*<0.01 Additional control variables: survey effects.

**References**

Aron, A., E. N. Aron and D. Smollan (1992). "Inclusion of other in the self scale and the structure of interpersonal closeness." Journal of Personality and Social psychology **63**(4): 596-612.

Bruchon-Schweitzer, M. and I. Paulhan (1993). Adaptation francophone de l’inventaire d’anxiété Trait-Etat (Forme Y) de Spielberger. Paris, France, Editions du Centre Psychologie Appliquée.

Cheek, J. M., S. Smith and L. R. Tropp (2002). Relational identity orientation: A fourth scale for the AIQ. Annual Meeting of the Society for Personality and Social Psychology, Savannah, GA, Savannah, CA.

Cheek, N. N., J. M. J. S. Cheek and Identity (2018). "Aspects of identity: From the inner-outer metaphor to a tetrapartite model of the self." Self and Identity **17**(4): 467-482.

Dormont, B., A.-L. Samson, M. Fleurbaey, S. Luchini and E. Schokkaert (2018). "Individual Uncertainty About Longevity." Demography **55**(5): 1829-1854.

Ersner-Hershfield, H., M. T. Garton, K. Ballard, G. R. Samanez-Larkin and B. Knutson (2009). "Don’t stop thinking about tomorrow: Individual differences in future self-continuity account for saving." Judgment and Decision Making **4**(4): 280-286.

Falk, A., A. Becker, T. Dohmen, B. Enke, D. Huffman and U. Sunde (2018). "Global evidence on economic preferences." The Quarterly Journal of Economics **133**(4): 1645-1692.

Gauthier, J. and S. Bouchard (1993). "Adaptation canadienne-française de la forme révisée du State–Trait Anxiety Inventory de Spielberger." Canadian Journal of Behavioural Science/Revue canadienne des sciences du comportement **25**(4): 559-578.

Hershfield, H. E. (2011). "Future self-continuity: How conceptions of the future self transform intertemporal choice." Annals of the New York Academy of Sciences **1235**: 30-43.

Marteau, T. M. and H. Bekker (1992). "The development of a six‐item short‐form of the state scale of the Spielberger State—Trait Anxiety Inventory (STAI)." British journal of clinical Psychology **31**(3): 301-306.

Yin, R. and F. Étilé (2019). "Mesurer les orientations de l’identité pour comprendre les préférences." Revue Economique **70**(6): 1053-1078.
